# CSF1R inhibition with PLX5622 affects multiple immune cell compartments and induces tissue-specific metabolic effects in lean mice

**DOI:** 10.1007/s00125-023-06007-1

**Published:** 2023-10-04

**Authors:** Angela J. T. Bosch, Lena Keller, Laura Steiger, Theresa V. Rohm, Sophia J. Wiedemann, Andy J. Y. Low, Marc Stawiski, Leila Rachid, Julien Roux, Daniel Konrad, Stephan Wueest, Sonia Tugues, Melanie Greter, Marianne Böni-Schnetzler, Daniel T. Meier, Claudia Cavelti-Weder

**Affiliations:** 1 Department of Biomedicine, University of Basel, Basel, Switzerland; 2 Swiss Institute of Bioinformatics, Basel, Switzerland; 3 Division of Pediatric Endocrinology and Diabetology, University Children’s Hospital, University of Zurich, Zurich, Switzerland; 4 Children’s Research Centre, University Children’s Hospital, University of Zurich, Zurich, Switzerland; 5 Institute of Experimental Immunology, University of Zurich, Zurich, Switzerland; 6 Department of Endocrinology, Diabetology and Clinical Nutrition, University Hospital Zurich (USZ), University of Zurich (UZH), Zurich, Switzerland

**Keywords:** Colony stimulating factor 1 (CSF1), Eosinophils, IL-1β, Innate lymphoid cells, Insulin secretion, Insulin sensitivity, Macrophages, PLX5622

## Abstract

**Aims/hypothesis:**

Colony stimulating factor 1 (CSF1) promotes the proliferation, differentiation and survival of macrophages, which have been implicated in both beneficial and detrimental effects on glucose metabolism. However, the physiological role of CSF1 signalling in glucose homeostasis and the potential therapeutic implications of modulating this pathway are not known. We aimed to study the composition of tissue macrophages (and other immune cells) following CSF1 receptor (CSF1R) inhibition and elucidate the metabolic consequences of CSF1R inhibition.

**Methods:**

We assessed immune cell populations in various organs by flow cytometry, and tissue-specific metabolic effects by hyperinsulinaemic–euglycaemic clamps and insulin secretion assays in mice fed a chow diet containing PLX5622 (a CSF1R inhibitor) or a control diet.

**Results:**

CSF1R inhibition depleted macrophages in multiple tissues while simultaneously increasing eosinophils and group 2 innate lymphoid cells. These immunological changes were consistent across different organs and were sex independent and reversible after cessation of the PLX5622. CSF1R inhibition improved hepatic insulin sensitivity but concomitantly impaired insulin secretion. In healthy islets, we found a high frequency of IL-1β^+^ islet macrophages. Their depletion by CSF1R inhibition led to downregulation of macrophage-related pathways and mediators of cytokine activity, including *Nlrp3*, suggesting IL-1β as a candidate insulin secretagogue. Partial restoration of physiological insulin secretion was achieved by injecting recombinant IL-1β prior to glucose stimulation in mice lacking macrophages.

**Conclusions/interpretation:**

Macrophages and macrophage-derived factors, such as IL-1β, play an important role in physiological insulin secretion. A better understanding of the tissue-specific effects of CSF1R inhibition on immune cells and glucose homeostasis is crucial for the development of targeted immune-modulatory treatments in metabolic disease.

**Data availability:**

The RNA-Seq dataset is available in the Gene Expression Omnibus (GEO) under the accession number GSE189434 (http://www.ncbi.nlm.nih.gov/geo/query/acc.cgi?acc=GSE189434).

**Graphical Abstract:**

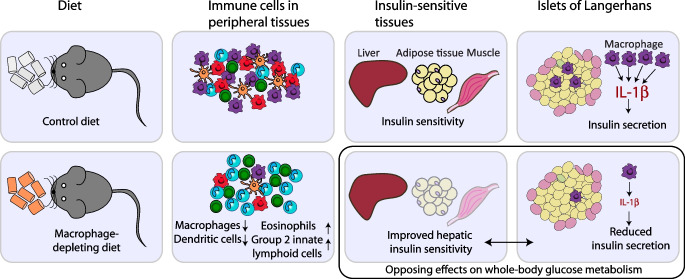

**Supplementary Information:**

The online version of this article (10.1007/s00125-023-06007-1) contains peer-reviewed but unedited supplementary material.


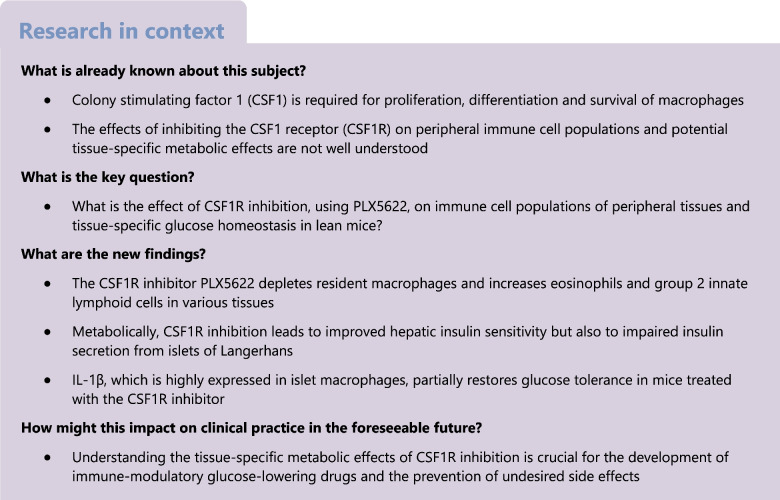


## Introduction

Colony stimulating factor 1 (CSF1), also known as macrophage colony stimulating factor (M-CSF), has a pivotal role in promoting proliferation, differentiation and survival of macrophages [1, 2]. Moreover, CSF1 regulates macrophage activation *in vitro* and inflammatory responses *in vivo* [3]. The CSF1 receptor (CSF1R) is expressed on cells of the mononuclear phagocyte system [2, 4]. In mice, a spontaneous mutation of the *Csf1* gene or a knockout of its receptor *Csf1r* leads to macrophage deficiency in most tissues [5, 6]. CSF1R can be pharmacologically targeted by either CSF1R antibodies or inhibitors [7, 8]. The small molecule PLX5622, a highly selective inhibitor of CSF1R kinase, has been used to deplete microglia and, thereby, alter energy intake and expenditure in high-fat-diet (HFD)-fed mice [9, 10]. Recent data suggest that the effect of PLX5622 is not restricted to microglia and that this agent also targets myeloid compartments in the bone marrow and spleen [11]. Manipulation of CSF1 signalling via small molecule inhibitors, antibodies, or mutations also leads to effects on myeloid cells outside the brain [2]. However, a comprehensive view of the effects of CSF1R inhibition on peripheral immune cell populations and potential metabolic consequences is currently lacking.

Macrophages play a key role in both insulin resistance and beta cell dysfunction, the two main drivers of type 2 diabetes. Accumulation of macrophages and activation of proinflammatory pathways in insulin target tissues such as adipose tissue [12, 13], liver [14] and muscle [15] suggest a role of tissue macrophages in promoting insulin resistance. Conversely, macrophages within the islets of Langerhans exert beneficial effects on beta cell mass and function. During islet development, macrophages are shown to be critical for beta cell formation as mice deficient for *Csf1* have impaired beta cell development and reduced beta cell mass [16, 17]. In injury models, islet macrophages have been shown to support beta cell replication and enhance glucose-stimulated insulin secretion (GSIS) by shifting beta cells towards a reparative state [18–20]. Conversely, islet macrophages accumulate during the development of type 2 diabetes, resulting in deleterious effects on insulin secretion [21–23]. Hence, there seems to be a finely tuned balance between islet macrophages and beta cells, which can be disturbed by macrophage accumulation or prolonged immune cell activation.

Since tissue-resident macrophages exert crucial roles in glucose homeostasis, we aimed to study the composition of tissue macrophages (and other immune cells) following CSF1R inhibition using PLX5622 and the metabolic consequences of this inhibition. A better understanding of how tissue-resident macrophages in different organs contribute to glucose homeostasis is crucial for the advancement of targeted immune-modulatory treatments in type 2 diabetes and metabolic disease.

## Methods

### Mice

Wild-type C57BL/6NCrl mice were originally purchased from Charles River laboratories (Germany; RRID:IMSR_CRL:027; http://www.criver.com/products-services/find-model/c57bl6-mouse?region=3616), bred in-house and maintained under specific pathogen-free conditions with free access to water and food in a 12 h dark–light cycle. Experiments were conducted in male mice, except for reversibility studies. Mice were assigned to weight-matched groups. Blinding was not feasible during treatment; however, results were analysed in a blinded fashion whenever possible. Data were not included if misinjection occurred during GTT, or if values were excluded by outlier test. All animal experiments were done according to the Swiss veterinary law and institutional guidelines and approved by Swiss authorities.

### Pharmacological depletion of macrophages

Mice were fed a diet containing the CSF1R inhibitor PLX5622 (provided by Plexxikon, USA) at a concentration of 1200 ppm (estimated mean plasma concentration of 9090 ng/ml [10]) or were fed the corresponding control diet (AIN76; Research Diets, USA) from 4–5 weeks of age (long-term) or 8–10 weeks of age (3–5 weeks’ exposure) until euthanasia. For reversibility studies, mice were fed a PLX5622-supplemented diet for 4 weeks, followed by control diet for 3–4 weeks. Alternatively, mice received 1 mg/mouse anti-mouse CD115 antibody (AFS98; BioXCell, USA) or rat IgG2a isotype control (2A3; BioXCell) in saline (154 mmol/l NaCl) by weekly i.p. injection for 3 weeks. The dosage was adapted from previous literature [24]. Ex vivo macrophage depletion was achieved by culturing islets with clodronate or control PBS liposomes for 24 h, as described previously [23].

### GTT

Mice were fasted for 6 h and given an i.p. injection of glucose (2 g/kg body weight; B. Braun, Germany). Blood glucose was measured at 0, 15, 30, 60, 90 and 120 min and blood was collected at 0, 15 and 30 min for insulin measurements. Recombinant IL-1β (1 µg/kg; R&D Systems, USA) or saline (154 mmol/l NaCl) was injected 18 min prior to the GTT, as described previously [25].

### Hyperinsulinaemic–euglycaemic clamps

A catheter (MRE 025; Braintree Scientific, USA) was inserted into the right jugular vein and exteriorised at the neck. Hyperinsulinaemic–euglycaemic clamps were performed 5–7 days after surgery in freely moving mice after 5 h of fasting. To assess basal glucose turnover, mice were infused with 3-[^3^H]glucose (Perkin Elmer, USA) for 80 min. Hyperinsulinaemia was induced by a constant insulin infusion paralleled by 3-[^3^H]glucose infusion. Steady-state glucose infusion rate (GIR) was calculated once glucose infusion reached a constant rate for 15–20 min, with blood glucose levels clamped at ~5–6 mmol/l. To assess tissue-specific glucose uptake, a bolus of 2-deoxy[1-^14^C]glucose (Perkin Elmer) was administered via catheter at the end of the steady-state period. Blood was sampled at 2, 15, 25 and 35 min thereafter. For details, see electronic supplementary material (ESM) Methods.

### Isolation and flow cytometry of immune cells

Immune cells from adipose tissue, liver and pancreas were isolated by collagenase IV (Worthington, USA) digestion, while colon tissue was digested by collagenase VIII (Sigma Aldrich, USA) and lung tissue using liberase (Roche, Switzerland). Microglia and spleen were mechanistically dissociated. Peritoneal cells were isolated by abdominal lavage. Blood immune cells were isolated using BD FACS lysis solution (BD, USA). To isolate islet immune cells, islets were digested using trypsin-EDTA (0.5%; Gibco, USA), and immune cells were cultured for 4 h in 2 mmol/l or 20 mmol/l glucose in the presence of Brefeldin A (5 µg/ml; Sigma) and Monensin (1 µg/ml; Sigma). For details, see ESM Methods and ESM Table 1. Gating strategies are shown in ESM Figs. 1 and 2.

### Gene expression analysis

RNA isolation was performed using the NucleoSpin kit (Macherey-Nagel, Germany) or the RNeasy Plus Universal Mini kit (Qiagen, Germany). Reverse transcription was carried out using GoScript (Promega, USA) and real-time PCR (ViiA7; Thermo Fisher Scientific, USA) with GoTaq qPCR Master Mix (Promega). Primers were ordered from Microsynth (Switzerland); for primer sequences see ESM Table 2.

### Protein expression analysis

Plasma insulin, TNF, IL-6, granulocyte-macrophage colony-stimulating factor (GM-CSF), IL-33, IL-5 and IL-13 were quantified by electrochemiluminescence (MESO SECTOR S 600) using kits from Meso Scale Discovery (USA).

### Liver enzymes and lipids

Liver enzymes and blood lipids were measured in plasma on the c502/c702 modules of the Cobas 8000 series (Roche Diagnostics, Switzerland).

### GSIS

To measure GSIS, mouse islets were isolated by collagenase digestion (1.5 mg/ml Collagenase IV) and purified by filtration and hand-picking. Islets were cultured overnight, free-floating in RPMI-1640 medium containing 11.0 mmol/l glucose, 100 U/ml penicillin, 100 μg/ml streptomycin, 2 mmol/l glutamax, 50 μg/ml gentamicin, 0.25 μg/ml amphotericin B and 10% (vol./vol.) FCS, washed and then incubated in modified KRB (NaCl, KCl, CaCl_2_ 2H_2_O, KH_2_PO_4_, MgSO_4_ 7H_2_O) containing 2.8 or 16.7 mmol/l glucose and 0.5% (wt/vol.) BSA for 1 h. Islet insulin content was extracted with 0.18 mol/l HCl in ethanol.

### Beta cell mass

Pancreas sections were stained for insulin and CD45 (two per mouse, 100 µm apart), or insulin and glucagon. Beta cell mass was defined as the ratio of insulin-positive area to total pancreas area multiplied by pancreas weight. Analysis was performed using Fiji software in a semi-automated way (ImageJ 2.3.0/1.53q; Java 1.8.0_265 [64-bit]; https://imagej.net/Fiji), using ilastik software (Version 1.3.2; http://www.ilastik.org/). For details, see ESM Methods and ESM Table 1.

### RNA sequencing

RNA was extracted from pancreatic islets and was quantified and quality-controlled by High Sensitivity RNA ScreenTape on an Agilent (USA) TapeStation instrument. Library preparation (Illumina Truseq stranded kit; Illumina, USA) was performed at the Genomics Facility Basel of the ETH Zurich, from 80 ng total RNA, and controlled on an Agilent Fragment Analyzer instrument. Sequencing was performed on the Illumina NextSeq 500 platform. Data analysis was performed by the Bioinformatics Core Facility, University of Basel (Switzerland). Read quality was assessed with FastQC (version 0.11.5). For details, see ESM Methods.

### Quantification and statistical analysis

Data are expressed as mean±SEM. The unpaired Mann–Whitney *U* test or two-way ANOVA was used to test statistical differences (GraphPad Prism, USA). A *p* value of <0.05 was considered statistically significant. GTT data show one representative experiment, all other data are pooled. RNAseq data was analysed using the R software (version 4.0.0; http://www.r-project.org) and the Bioconductor 3.11 packages (http://bioconductor.org/).

## Results

### CSF1R inhibition by PLX5622 depletes resident macrophages in multiple organs

**Fig. 1:**
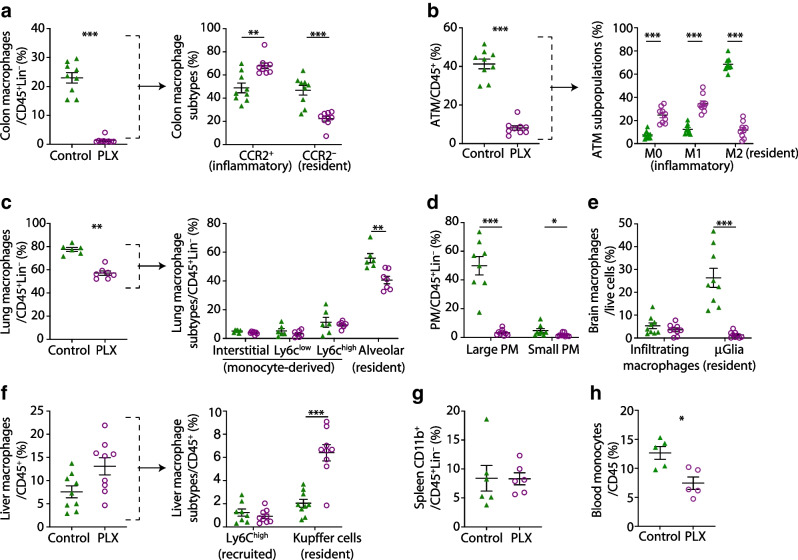
CSF1R inhibition by PLX5622 depletes resident macrophages in multiple organs. Male wild-type mice were fed a PLX5622-containing diet (purple circles) or control diet (green triangles) from 4–5 weeks of age onwards, for up to 5.5 months (except for blood analysis, whereby treatment started at 9–10 weeks of age for 3 weeks). Frequencies of total macrophages are shown for (**a**) the colon, (**b**) adipose tissue, (**c**) the lung, (**d**) the peritoneum, (**e**) the brain, (**f**) the liver, (**g**) the spleen and (**h**) monocytes in the blood. Frequencies of macrophage subtypes are also shown for the colon (**a**), adipose tissue (**b**), the lung (**c**) and the liver (**f**). For gating strategies, see ESM Fig. 1. Data are shown as mean±SEM and representative of four (**h**), three (**a**, **b**, **f**), two (**d**, **e**, **g**) or one (**c**) independent experiment(s), with each data point representing an individual mouse. ATM, adipose tissue macrophages; CCR2, C-C motif chemokine receptor 2; PLX, PLX5622; PM, peritoneal macrophages; μGlia, microglia; Lin, lineage (‘dump channel’, including CD3-, CD19-, Ly6G- and NK1.1-specific antibodies to exclude T and B cells, granulocytes and natural killer cells). *p<0.05, **p<0.01, ***p<0.001, analysed by unpaired Mann–Whitney *U* test with two-tailed distribution

To assess the effects of CSF1R inhibition on tissue macrophages, we analysed macrophage subpopulations in multiple organs of male wild-type mice fed a diet supplemented with or without the CSF1R inhibitor PLX5622. After 5 months, we found a 95% reduction in the frequency of microglia as reported [9] and decreased macrophages in the colon (92.4%), adipose tissue (58.2%), lung (26.3%) and peritoneal cavity (90.2%) (Fig. 1a–d). CSF1R inhibition predominantly depleted tissue-resident-type macrophages like C-C motif chemokine receptor 2 (CCR2)^−^ colon macrophages, CD206^+^ M2 adipose tissue macrophages, Siglec-F^+^ alveolar macrophages, F4/80^high^CD11b^high^ large peritoneal macrophages and microglia (Fig. 1a–e). Absolute numbers were reduced in colon and adipose tissue but not in the lungs (ESM Fig. 3). Kupffer cells in the liver were increased in frequency but recruited Ly6C^high^ macrophages were unchanged (Fig. 1f). Splenic CD11b^+^ cells, including monocytes and eosinophils, were not affected by PLX5622, while blood monocytes were reduced in PLX5622-treated mice (23.1% reduction) (Fig. 1g,h).

To investigate whether the degree of macrophage depletion is influenced by CSF1R expression, we analysed two previously published RNA-Seq datasets of different macrophage populations [26, 27]. Microglia exhibited the highest expression of CSF1R, correlating with our observation that this was the most significantly reduced tissue macrophage subpopulation following CSF1R inhibition. Macrophages from the colon, peritoneum and adipose tissue demonstrated ‘intermediate’ levels of CSF1R expression, consistent with a high-to-intermediate depletion with CSF1R inhibition. Lung and monocytes exhibited the lowest CSF1R expression levels, in line with the lowest depletion efficiency (ESM Fig. 4). Thus, CSF1R inhibition by PLX5622 resulted in macrophage depletion in different tissues, primarily affecting resident macrophages, depending on CSF1R expression levels.

### CSF1R inhibition increases eosinophils and group 2 innate lymphoid cells and decreases CD11b^+^ dendritic cells

**Fig. 2:**
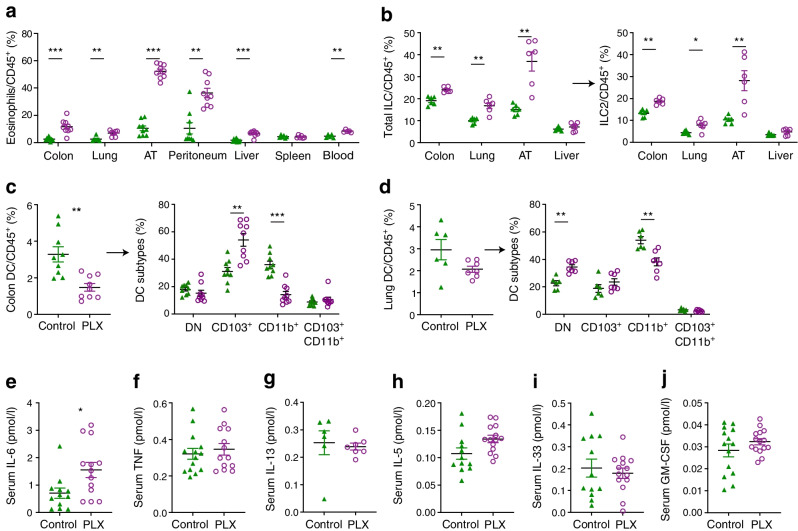
CSF1R inhibition increases eosinophils and ILC2 and decreases CD11b^+^ DCs. Male wild-type mice were fed a PLX5622-containing diet (purple circles) or control diet (green triangles) from 4–5 weeks of age onwards, for up to 5.5 months (except for innate lymphoid cell [ILC], blood analysis and serum cytokine measurements, whereby diets started at 9–10 weeks of age for 3 weeks). (**a**) Frequency of eosinophils gated on CD45^+^ cells in the colon, lung, adipose tissue, peritoneum, liver, spleen and blood. (**b**) Frequency of ILCs and ILC2s gated on CD45^+^ cells in the colon, lung adipose tissue and liver. (**c**, **d**) Total DCs and their subtypes in the colon (**c**) and lung (**d**). (**e**–**j**) Serum IL-6 (**e**), TNF (**f**), IL-13 (**g**), IL-5 (**h**), IL-33 (**i**) and GM-CSF (**j**). For gating strategies see ESM Fig. 1 and ESM Fig. 2. Data are shown as mean±SEM and are representative of three (**a** [colon, AT, liver], **b**, **c**), two (**a** [peritoneum, spleen, blood]) or one (**a** [lung], **d**) independent experiment(s), or pooled data from two (**e**, **f**) and three independent experiments (**g**–**j**), with each data point representing an individual mouse. AT, adipose tissue; DN, double negative; PLX, PLX5622. *p<0.05, **p<0.01, ***p<0.001, analysed by unpaired Mann–Whitney *U* test with two-tailed distribution

Whether CSF1R inhibition modulates immune cells in peripheral tissues other than macrophages is currently not well known. Upon PLX5622 treatment, frequencies of eosinophils were consistently increased in the colon (350.1%), lung (174.9%), adipose tissue (399.5%), peritoneal cavity (248.0%), liver (279.8%) and blood (77.5%) of the mice, but were not increased in the spleen (Fig. 2a). In parallel, group 2 innate lymphoid cells (ILC2s) were elevated in the colon (41%), lung (43.7%) and adipose tissue (173%, Fig. 2b). In addition, we found that CD11b^+^ dendritic cells (DCs) were significantly reduced in the colon (60.5%) and lung (41.0%) (Fig. 2c,d). Other immune cells, such as B and T lymphocytes, natural killer cells and neutrophils, did not show a consistent pattern across different tissues (ESM Fig. 5). T cell development in the thymus was unaffected by PLX5622 treatment (ESM Fig. 6). Circulating plasma IL-6 was increased, while TNF, IL-13, IL-5, IL-33 and GM-CSF remained unchanged (Fig. 2e–j). Hence, besides effects on tissue-resident macrophages, CSF1R inhibition by PLX5622 led to increased eosinophils and ILC2s and reduced CD11b^+^ DCs across multiple organs.

### Immune cell alterations revert after cessation of CSF1R inhibition

**Fig. 3:**
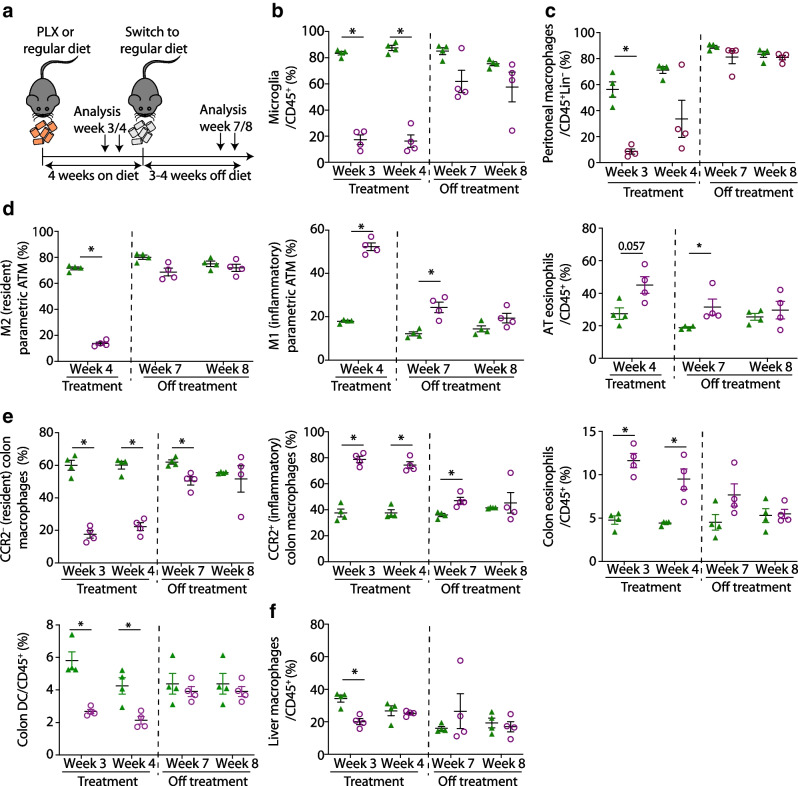
Immune cell alterations revert after cessation of CSF1R inhibition. (**a**) Schematic illustration of the experimental set-up in female wild-type mice fed a PLX5622-containing diet or control diet starting at 6–7 weeks of age. After 4 weeks, all mice received control diet. (**b**–**f**) Frequency of microglia in brain (**b**), macrophages in the peritoneum (**c**), M2 (resident) macrophages, M1 (inflammatory) macrophages and eosinophils in adipose tissue (**d**), C-C motif chemokine receptor 2 (CCR2)^−^ (resident) macrophages, CCR2^+^ (inflammatory) macrophages, eosinophils and DCs in the colon (**e**) and liver macrophages (**f**) in mice originally fed a PLX5622-containing diet (purple circles) or control diet (green triangles). Data are shown as mean±SEM of one experiment with four mice each experiment; each data point represents an individual mouse. AT, adipose tissue; ATM, adipose tissue macrophages; Lin, lineage (‘dump channel’, including CD3-, CD19-, Ly6G- and NK1.1-specific antibodies to exclude T and B cells, granulocytes and natural killer cells); PLX, PLX5622. *p<0.05, analysed by unpaired Mann–Whitney *U* test with two-tailed distribution

Repopulation of microglia has been shown to occur after removal of PLX5622 [8]. Also in bone marrow and spleen, myeloid and lymphoid cell populations recovered after withdrawal of PLX5622 [11]. To address sex-specific effects as well as reversibility of the phenotype, we treated female wild-type mice with PLX5622 for 4 weeks, followed by 3–4 weeks of control diet prior to characterisation of immune cells (Fig. 3a). Similar to male mice, tissue-resident macrophages in female mice were depleted after 3–4 weeks of PLX5622, while DCs were reduced and eosinophils were elevated (Fig. 3b–e). At 3–4 weeks after cessation of PLX5622, peritoneal macrophages, microglia and tissue-resident macrophages of adipose tissue and colon recovered and the relative increase in inflammatory macrophages returned to baseline levels (Fig. 3b–e). Colon and adipose tissue eosinophils and colon DCs were also restored after PLX5622 was stopped (Fig. 3d,e). The withdrawal of PLX5622 did not affect the abundance of macrophages in liver tissue (Fig. 3f). In sum, PLX5622-induced immune cell alterations in peripheral tissues were reversible after cessation of CSF1R inhibition and were sex independent.

### Immune cell alterations are directly dependent on CSF1R inhibition

**Fig. 4:**
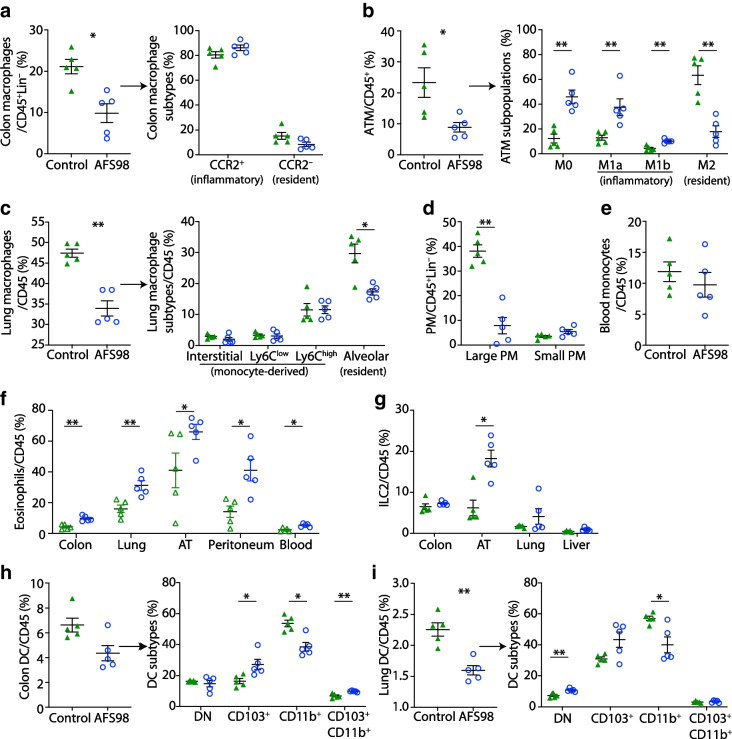
Immune cell alterations are directly dependent on CSF1R inhibition, as CSF1R antibody results in the same immune cell alterations as treatment with PLX5622. Wild-type mice were treated once weekly with 1 mg of the CSF1R antibody AFS98 (blue circles) or rat IgG2a isotype control (green triangles) starting from 9 weeks of age for 3 weeks. (**a**–**e**) Frequencies of total macrophages in the colon (**a**), adipose tissue (**b**), lung (**c**), peritoneum (**d**) and blood monocytes (**e**). Frequencies of macrophage subtypes are also shown for the colon (**a**), adipose tissue (**b**) and lung (**c**). (**f**, **g**) Frequencies of eosinophils in the colon, lung, adipose tissue, peritoneum and blood (**f**) and of ILC2s in the colon, adipose tissue, lung and liver (**g**). (**h**, **i**) Frequencies of DCs and their subtypes in the colon (**h**) and lung (**i**). Data are shown as mean±SEM of one experiment with five mice each. AT, adipose tissue; ATM, adipose tissue macrophages; CCR2, C-C motif chemokine receptor 2; DN, double negative; Lin, lineage (‘dump channel’, including CD3-, CD19-, Ly6G- and NK1.1-specific antibodies to exclude T and B cells, granulocytes and natural killer cells); PM, peritoneal macrophages. *p<0.05, **p<0.01, analysed by unpaired Mann–Whitney *U* test with two-tailed distribution

Next, we assessed whether the immune cell phenotype was driven by CSF1R inhibition or by PLX5622-specific off-target effects such as tyrosine kinase inhibition. Mice were treated once weekly with a monoclonal CSF1R antibody (AFS98) or an isotype control and immune cells were characterised after 3 weeks [24]. CSF1R antibody treatment reduced macrophage frequencies across multiple tissues (reduction of 53.5% in colon, 61.9% in adipose tissue, 28.5% in lung and 67.7% in peritoneal cavity), particularly tissue-resident subpopulations (i.e. CD206^+^ M2 macrophages, Siglec-F^+^ alveolar macrophages and F4/80^high^CD11b^high^ peritoneal macrophages) (Fig. 4a–d and ESM Fig. 7). Blood monocytes were not reduced by AFS98 treatment (Fig. 4e). Similar to CSF1R inhibition by PLX5622, eosinophils and ILC2s were elevated in the colon, lung, adipose tissue, peritoneal cavity and blood, while CD11b^+^ DCs were decreased in the colon and lung (Fig. 4f–i and ESM Fig. 8). In sum, the immune cell phenotype upon CSF1R inhibition by PLX5622 was recapitulated with the neutralising CSF1R antibody AFS98, thus excluding the possibility of PLX5622-specific off-target effects.

### CSF1R inhibition leads to improved hepatic insulin sensitivity

**Fig. 5:**
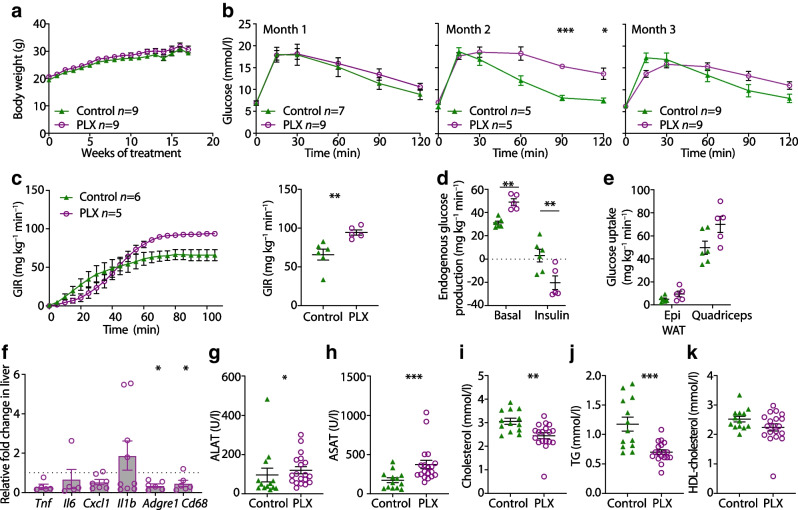
CSF1R inhibition leads to improved hepatic insulin sensitivity. Male wild-type mice were fed a PLX5622-containing diet (purple circles) or control diet (green triangles) from 4–5 weeks of age onwards, for up to 5.5 months. (**a**, **b**) Time course of body weight (**a**) and glucose tolerance (**b**) in male mice fed control or PLX5622 diet (two-way ANOVA, *p*=0.008, comparing PLX5622 with control at 2 months). (**c**) Hyperinsulinaemic–euglycaemic clamps in male mice fed a control or PLX5622-containing diet for 4 months, showing GIR over time and at steady state. (**d**) Endogenous glucose production in the liver. (**e**) Glucose uptake into epididymal white adipose tissue and skeletal muscles (quadriceps). (**f**) qPCR analysis of inflammatory and macrophage markers in liver tissue, comparing mice fed a PLX5622-containing diet with those fed a control diet (*n*=9 for control diet; dotted line represents 1 [i.e. no change]). (**g**–**k**) Plasma analysis of the liver enzymes alanine aminotransferase (**g**) and aspartate aminotransferase (**h**), cholesterol (**i**), triacylglycerol (**j**) and HDL-cholesterol (**k**) after 4–5 months of being fed a control diet or PLX5622-containing diet. Data are shown as mean±SEM and representative of three (**a**, **b**, **f**–**k**) or one independent experiments (**c**–**e**). For all graphs showing single data points (**c–k**), each data point represents an individual mouse; all other graphs (**a–c**) show pooled data. ALAT, alanine aminotransferase; ASAT, aspartate aminotransferase, Epi WAT, epididymal white adipose tissue; PLX, PLX5622; TG, triacylglycerol. *p<0.05, **p<0.01, ***p<0.001, unpaired Mann–Whitney *U* test with two-tailed distribution

Insulin resistance has been related to macrophage accumulation in peripheral tissues [28]. We assessed whether macrophage depletion upon CSF1R inhibition in turn improves glucose homeostasis. Body weights of PLX5622-treated male mice and controls did not differ (Fig. 5a). After 3 months of treatment, glucose tolerance was improved after 15 min but was impaired after 120 min in PLX5622-treated mice (Fig. 5b).

To assess tissue-specific insulin sensitivity, we performed hyperinsulinaemic–euglycaemic clamps in mice fed PLX5622 for 4 months. An increased GIR indicated improved whole-body insulin sensitivity (Fig. 5c). Endogenous glucose production (reflecting hepatic glucose production) was elevated in PLX5622-treated mice basally but was significantly reduced under hyperinsulinaemic conditions (Fig. 5d). Glucose uptake into adipose tissue did not differ between PLX5622-treated and control groups, whereas glucose uptake into skeletal muscle was increased in PLX5622-treated mice, yet the difference did not reach statistical significance (*p*=0.0519; Fig. 5e). Hepatic gene expression of macrophage markers and proinflammatory cytokines, as well as systemic cholesterol, HDL-cholesterol and triacylglycerol (TG) were reduced and alanine aminotransferase (ALAT) and aspartate aminotransferase (ASAT) were elevated in mice treated with PLX5622 compared with controls (Fig. 5f–k). Hence, CSF1R inhibition by PLX5622 led to a discordant behaviour of impaired glucose tolerance but improved insulin sensitivity.

### Macrophage depletion by CSF1R inhibition leads to impaired beta cell function

**Fig. 6:**
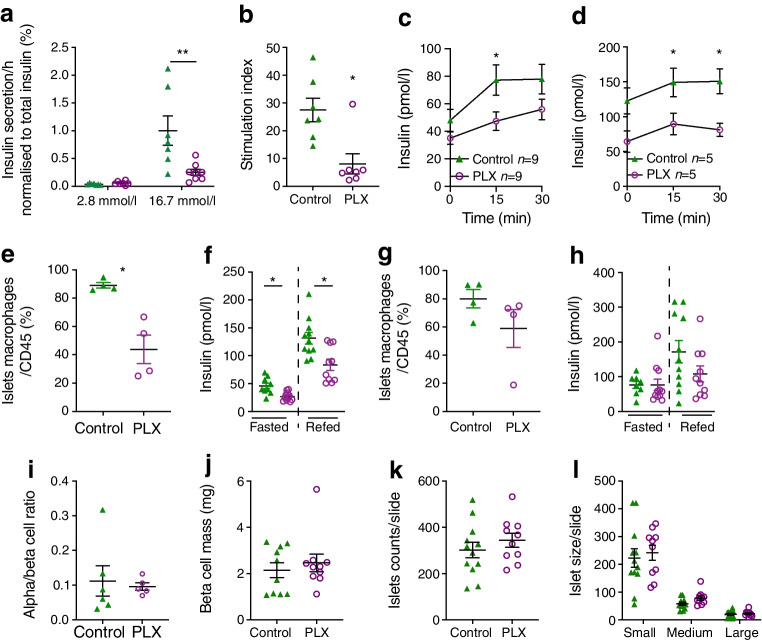
Macrophage depletion following CSF1R inhibition leads to impaired beta cell function. Male wild-type mice were fed a PLX5622-containing diet (purple circles) or control diet (green triangles) from 4–5 weeks of age onwards, for up to 8 months. (**a**, **b**) Ex vivo GSIS (**a**) and stimulation index (**b**) after 5.5 months of PLX5622 treatment. (**c**, **d**) In vivo insulin secretion upon glucose stimulation after 3 months (**c**) and 8 months (**d**) of treatment, comparing control with PLX5622-treated mice. (**e**–**h**) Islet macrophages (**e**, **g**) and insulin secretion (**f**, **h**) in female mice upon fasting and refeeding after 4 weeks on (**e**, **f**) and 4 weeks off (**g**, **h**) PLX5622 treatment. (**i**–**l**) Alpha/beta cell ratio (**i**), beta cell mass (**j**), number of islets (**k**) and size distribution (small, 10–1000 µm^2^; medium, 1000–10,000 µm^2^; large, 10,000–100,000 µm^2^) (**l**). Data are shown as mean±SEM and representative of three (**a**, **b**), two (**c**, **d**, **i**–**l**) or one independent experiment(s) (**e**–**h**). For all graphs showing single data points (**a**, **b**, **e–h**, **j–l**), each data point represents an individual mouse. PLX, PLX5622. *p<0.05, **p<0.01, unpaired Mann–Whitney *U* test with two-tailed distribution

The discordant behaviour of impaired glucose tolerance alongside improved insulin sensitivity in PLX5622-treated mice suggested that CSF1R inhibition affected beta cell function. To exclude a toxic effect on beta cells, we treated wild-type mouse islets for 72 h with PLX5622 in vitro. PLX5622 did not lead to reduced insulin secretion or islet macrophage depletion in vitro (ESM Fig. 9). Islets isolated from mice fed PLX5622 for 5.5 months, however, showed impaired insulin secretion ex vivo (Fig. 6a,b), which was in line with impaired basal and glucose-stimulated insulin levels during the GTT in PLX5622-treated mice compared with controls as early as 1 month after treatment (Fig. 6c,d). Comparing the number of islet macrophages with beta cell function in vivo, we found a 50% reduction in islet macrophage numbers in mice treated for 4 weeks with PLX5622, together with a functional beta cell defect as indicated by reduced insulin secretion (Fig. 6e,f). Four weeks after cessation of PLX5622 diet, the number of islet macrophages recovered and insulin secretion was no longer impaired (Fig. 6g,h).

To confirm these results, we assessed insulin secretion in two additional models of islet macrophage depletion. Treatment of mouse islets with clodronate liposomes in vitro led to an islet macrophage depletion of about 50%, which was accompanied by islet cell dysfunction as evidenced by slightly higher basal insulin release and diminished stimulated insulin secretion (ESM Fig. 10a, b), consistent with the current literature [23]. In addition, 3 weeks of treatment with AFS98 antibody in vivo led to an islet macrophage depletion of 53.5% and an insulin secretory defect (ESM Fig. 10c, d), confirming the negative effect of macrophage depletion on physiological insulin secretion.

Alpha/beta cell ratios, beta cell mass and islet cell size distribution in pancreases of mice treated for 5–6 months with PLX5622 or control diet were comparable (Fig. 6i–l). Hence, it is suggested that CSF1R inhibition by PLX5622 led to depletion of macrophages in islets and impaired beta cell function but not to reduced beta cell mass.

### Impaired beta cell function upon CSF1R inhibition can be partially restored by IL-1β

**Fig. 7:**
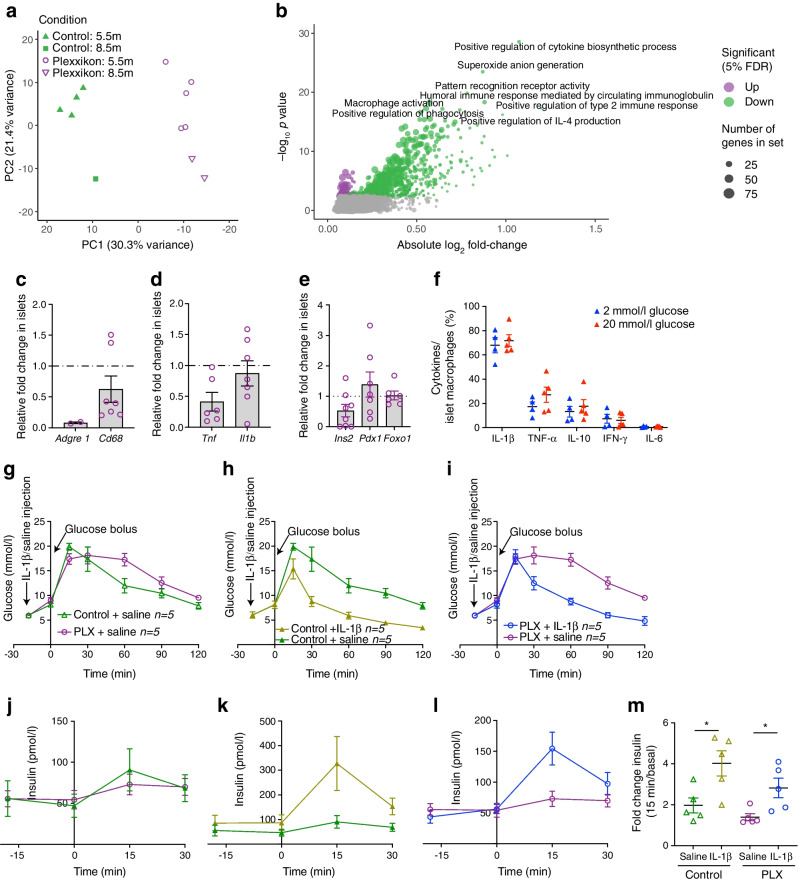
Impaired beta cell function upon CSF1R inhibition can be partially restored by IL-1β. (**a–e**) Male wild-type mice were fed a PLX5622-containing diet or control diet from 4–5 weeks of age onwards, for up to 8.5 months. (**a**) Principal component (PC) analysis of normalised expression levels across the RNA-Seq samples of mice treated for either 5.5 months (5.5m) or 8.5 months (8.5m). (**b**) Gene set enrichment analysis of the C5 MSigDB collection (see ESM Table 4), performed on results of the differential expression analysis comparing islets isolated from mice fed a PLX5622-containing diet (*n*=7) or control diet (*n*=6); terms were considered significant if their associated false discovery rate (FDR) was below 5%. (**c**–**e**) Expression of immune cell markers and beta cell identity genes in islets from PLX5622-treated mice relative to control mice (*n*=10 for control diet; dotted line represents 1 [i.e. no change]). (**f**) Flow cytometry analysis of cytokine expression in islets macrophages incubated in 2 mmol/l or 20 mmol/l glucose. Cells were gated for macrophages using CD45, F4/80 and CD11b and macrophages were analysed for expression of IL-1β, TNF-α, IL-10, IFN-γ and IL-6. (**g**–**m**) Male wild-type mice were fed a PLX5622-containing diet or control diet from 8–10 weeks of age onwards, for 5 weeks. Data was obtained after 4 and 5 weeks of PLX treatment in a crossover study, with a 1 week wash-out period. Glucose levels during GTT (**g**–**i**) after administration of IL1–1β or saline (at time point −18 min) and corresponding insulin secretion (**j**–**l**) under the following conditions: control+saline vs PLX+saline (**g**, **j**); control+IL-1β vs control+saline (**h**, **k**); and PLX+IL-1β vs PLX+saline (**i**, **l**). (**m**) Fold change in insulin during GTT. Data are shown as mean±SEM and are representative of two independent experiments (**a**–**f**) or one experiment (**g**–**m**). For all graphs showing single data points (**a**, **c–f**, **m**), each data point represents an individual mouse, while all other graphs (**b**, **g**–**l**) show pooled data. PLX, PLX6522. *p<0.05, analysed by unpaired Mann–Whitney *U* test with two-tailed distribution, or two-way ANOVA (GTT)

To identify potential pathways involved in beta cell dysfunction upon CSF1R inhibition, we performed RNA-Seq of islets isolated from mice treated with PLX5622 or control diet for 5.5 and 8.5 months. CSF1R inhibition separated clearly on the first component of a principal component analysis (Fig. 7a). When controlling for age and treatment duration, we found 284 genes differentially expressed in islets of mice on PLX5622 (5% false discovery rate [FDR]; ESM Table 3). Downregulated genes involved macrophage markers (*Adgre1*, *Cd86*, *Itgax, Ms4a7*, *Plbd1*), class 2 histocompatibility antigens (*H2-Aa*, *H2-Ab1*, *H2-Eb1*, *CD74*), complement components (*C1qb*, *C1qc*), lysosome-associated genes (*Laptm5*, *Ctss*) and genes involved in immune responses (*Mpeg1*, *Lilrb4a*, *Lilrb4b*, *Nckap1l*, *Cybb*, *Nlrp3*, *Tnf*), similar to the findings of a previous study with a CSF1R antibody [29] (ESM Fig. 11). This was mirrored by downregulated myeloid cell types (C8 cell-type signature gene sets) and dampened macrophage-related pathways in the gene set enrichment analysis (C5 gene ontology categories and Hallmark gene sets; Fig. 7b, ESM Fig. 12, and ESM Tables 4 and 5). Beta cell-related gene sets (i.e. Hallmark ‘pancreas beta cells’ and C8 cell-type gene set ‘muraro pancreas beta cells’ [see ESM Tables 5 and 6]) and transcription factors involved in beta cell differentiation (i.e. *Pdx*, *Mafa*, *Nkx2.2*, *Nkx6.1*, *Pax6*, *Neurod1*) were not differentially expressed upon CSF1R inhibition (ESM Table 6). We confirmed a non-significant reduction in gene expression of the macrophage/proinflammatory markers *Adgre1* (not detected in most samples from treated mice) and *Tnf* (*p*=0.48), and unchanged beta cell identity genes *Ins2*, *Foxo1 *and *Pdx1* in islets isolated from PLX5622-treated mice (Fig. 7c–e). In sum, beta cell dysfunction by CSF1R inhibition was associated with reduced macrophage markers and macrophage-related pathways in islets of Langerhans but not with dedifferentiated beta cells.

To address which macrophage-secreted factor could contribute to insulin secretion, we probed our RNA-Seq data for differential expression of mediators of cytokine activity. We found reduced expression of *Tnf*, *Nlrp3*, IFN-induced genes, *Ccl3*, and *Ccl5* in islets from mice treated with PLX5622 (ESM Table 7). To assess which cytokines could act as insulin secretagogues, we isolated islet macrophages from wild-type mice and assessed protein expression of different cytokines by flow cytometry. We found a high frequency of IL-1β^+^ islet macrophages, independent of the glycaemic condition (Fig. 7f). Due to IL-1β expression in islet macrophages and the IL-1β receptor being one of the most abundantly expressed cell surface receptors on mouse beta cells [30, 31], we tested the role of IL-1β in stimulating insulin secretion in mice devoid of macrophages. Administration of IL-1β prior to GTT in PLX5622-treated mice improved glucose tolerance and enhanced insulin secretion (Fig. 7 g–l): absolute insulin levels were lower but the fold increase (from basal to 15 min) was the same as that seen in controls (Fig. 7g–m), pointing towards a partial IL-1β-mediated effect on beta cell function. In summary, IL-1β derived from islet macrophages is an important, but not exclusive insulin secretagogue in pancreatic islets, promoting GSIS.

## Discussion

Our study provides a comprehensive analysis of the consequences of CSF1R inhibition on immune cells and its tissue-specific metabolic effects. We found profound changes in immune cells of myeloid and lymphoid origin upon CSF1R inhibition in various tissues. Especially intriguing is a reciprocal decrease in macrophages and CD11b^+^ DCs vs an increase in eosinophils and ILC2s across multiple tissues. Like macrophages, CD11b^+^ DCs express CSF1R, albeit at lower levels [4]. The increase in eosinophils and ILC2s could be compensatory after loss of resident macrophages. For example, immunological inhibitors secreted by resident macrophages limiting the expansion of eosinophils and ILC2s could be lacking (i.e. E-cadherin [32, 33]). ILC2s respond to IL-33 secreted from stromal cells and activate eosinophils via production of IL-5 and IL-13 [34]. Although circulating IL-33, IL-5 and IL-13 were not increased after treatment, local effects cannot be excluded. In addition, elevated CSF1 due to an increased compensatory production or decreased receptor-mediated endocytosis upon CSF1R blockade, as seen in CSF1R^−/−^ mice, could have off-target effects [5, 35].

These changes in immune cells were sex independent and reversible after withdrawal of the CSF1R inhibitor. The extent of macrophage depletion in different tissues may be explained by differential expression of CSF1R (i.e. macrophages with high CSF1R expression [microglia] were reduced to a higher degree than macrophages with low CSF1R expression [lung]). In the colon, with an intermediate level of CSF1R expression, the observed macrophage depletion was surprisingly robust. This suggests that additional factors, such as macrophage turnover, could contribute to the depletion efficiency.

An altered immune cell composition could affect tissue homeostasis and metabolism. We focused on the metabolic effects of CSF1 inhibition since tissue macrophages play a crucial role in insulin resistance and beta cell dysfunction. Previously, PLX5622 was shown to reduce peak glucose levels following glucose injection in chow-fed female mice [36]. Macrophage depletion by the CSF1R inhibitor PLX3397 did not influence glucose homeostasis in mice [37]. However, CSF1R inhibition by BLZ945 in mice fed HFD led to a dose-dependent macrophage reduction across multiple tissues, together with improved glucose tolerance and insulin sensitivity [38]. The strength of our study is that we conducted time course experiments to account for dynamic changes upon PLX5622 treatment. In chow-fed mice, we found a biphasic pattern during the GTT, with initially improved but later impaired whole-body glucose tolerance upon long-term PLX5622 treatment. Once present, this metabolic phenotype persisted under continued treatment. Several factors seem to dictate the metabolic response to CSF1R inhibition, such as the diet. While macrophage depletion is metabolically beneficial upon HFD feeding, as macrophage accumulation is reversed, its metabolic effects in a lean/healthy state are more complex. Due to opposing effects of macrophage depletion on insulin sensitivity (improved) and insulin secretion (impaired) in chow-fed mice, the metabolic effects counterbalance each other, making it challenging to discern any effects if tissue-specific readouts are not applied. It should also be noted that previous studies used female mice and employed a shorter duration of PLX5622 treatment [36, 37].

Tissue-specific metabolic readouts were key in unravelling the opposing metabolic effects of macrophage depletion. By hyperinsulinaemic–euglycaemic clamps, we found that CSF1R inhibition resulted in basally higher hepatic glucose production but enhanced insulin-induced suppression of gluconeogenesis, explaining the initially impaired and later improved whole-body insulin sensitivity observed in clamps. A similar effect on hepatic insulin sensitivity has been described upon macrophage depletion by clodronate liposomes [39, 40], implying it is connected to macrophage depletion rather than to CSF1R inhibition. Gene expression of inflammation and macrophage markers were reduced in liver tissue, suggesting an attenuated innate immune cell tone in the liver. Liver enzymes were elevated upon PLX5622 treatment, consistent with clinical studies using CSF1R inhibitors in cancer patients [41, 42], most likely as a consequence of decreased liver enzyme clearance by Kupffer cells [43].

While insulin sensitivity was initially impaired and then subsequently improved during clamps, glucose tolerance showed a reverse pattern, with an initially improved but later impaired glucose homeostasis. This discrepancy suggests that macrophage depletion has an independent effect on beta cell secretory function. Previously, islet macrophages have been shown to exert a beneficial effect on beta cell mass and function during development [16, 17] as well as on beta cell injury [18–20]. With long-standing macrophage accumulation in islets, as occurs in obesity and diabetes, the proinflammatory milieu has detrimental effects on beta cell function [21–23]. The physiological role of macrophages in adult/lean mice, however, is not clear. We found impaired GSIS by three different macrophage depletion models, while beta cell mass upon CSF1R inhibition was preserved.

Given the low abundance of immune cells within healthy islets, with approximately 5–7 cells per islet and 80% of these being macrophages [44], our investigation primarily focused on islet macrophages following CSF1R inhibition. The insulin secretion defect indeed correlated with the decrease in islet macrophages and was restored after cessation of the drug, indicative of a supportive role for islet macrophages in insulin secretion. RNA-Seq was used to address the pathways affected by macrophage depletion in islets. In accordance with a study using the CSF1R antibody AFS98 [29], macrophage markers and macrophage-related pathways were downregulated. Thereby, several mediators of cytokine activity were reduced in islets from PLX5622-treated mice, including TNF, nucleotide oligomerization domain-like receptor family pyrin domain-containing 3 (NLRP3) and IFN-related pathways. Due to the high abundance of IL-1β in islet macrophages, with its receptor known to be highly expressed on rodent beta cells [30, 31], IL-1β was the most promising candidate to impact on beta cell function. Previously, acute exposure to IL-1β in vivo has been shown to induce insulin secretion without changing insulin sensitivity in mice [25]. Conversely, blocking IL-1β decreased insulin secretion after food intake [25]. Older studies showed that gut-derived bacterial endotoxin is able to prime insulin secretion in rats [45]. We found that administration of IL-1β prior to glucose stimulation led to improved (albeit not completely restored) insulin secretion in macrophage-depleted mice, suggesting that IL-1β secreted by macrophages promotes physiological insulin secretion.

Our findings provide several insights with important clinical implications. First, we show that depletion of resident macrophages by CSF1R inhibition is associated with increased eosinophils and ILC2s across different tissues, indicating that immune cell populations are closely intertwined and depletion of one population may lead to compensatory changes in others. Second, macrophage depletion has tissue-specific metabolic effects such as improved hepatic insulin sensitivity, yet impaired beta cell function. Knowledge of the tissue-dependent metabolic effects of CSF1R inhibition is important in the development of immunomodulatory glucose-lowering drugs (e.g. liver-specific CSF1R inhibitors) or in the development of CSF1R inhibitors in cancer therapy [41]. More work is required to translate our results into therapeutic avenues for metabolic disease.

Taken together, our study shows that CSF1R inhibition leads to the depletion of resident tissue macrophages and a simultaneous increase in eosinophils and ILC2s and that these effects are reversible after cessation of the drug. These immune cell changes have tissue-specific metabolic effects, with improved hepatic insulin sensitivity and impaired beta cell function. Given the global burden of obesity and diabetes, a better understanding of tissue-specific effects of macrophages is of high clinical significance for the development of immune-modulatory therapeutics.

### Supplementary Information

Below is the link to the electronic supplementary material.

Supplementary file1 (PDF 1421 KB)

Supplementary file2 (XLSX 2837 KB)

## Data Availability

The RNA-Seq dataset is available in the Gene Expression Omnibus (GEO) under the accession number GSE189434 (http://www.ncbi.nlm.nih.gov/geo/query/acc.cgi?acc=GSE189434).

## Abbreviations

CSF1: Colony stimulating factor 1
CSF1R: Colony stimulating factor 1 receptor
DC: Dendritic cell
GM-CSF: Granulocyte-macrophage colony-stimulating factor
HFD: High-fat diet
ILC2: Group 2 innate lymphoid cells
