# Supplementary material for: CSF1R inhibition with PLX5622 affects multiple immune cell compartments and induces tissue-specific metabolic effects in lean mice
Source: Diabetologia. 2023 Oct 4;66(12):2292–306. doi: 10.1007/s00125-023-06007-1 (PMC10627931; doi:10.1007/s00125-023-06007-1)
Supplement: Supplementary file 1 — Supplementary file1 (PDF 1421 KB) [file 125_2023_6007_MOESM1_ESM.pdf]

Extended data

CSF1R-inhibition with PLX5622 affects multiple immune cell compartments and induces tissue-specific metabolic effects in lean mice

Immune cell and metabolic effects of CSF1R-inhibition

Authors: Angela J. T. Bosch[1], Lena Keller[1], Laura Steiger[1], Theresa V. Rohm[1], Sophia J. Wiedemann[1], Andy J. Y. Low[1], Marc Stawiski[1], Leila Rachid[1], Julien Roux[1,2], Daniel Konrad[3], Stephan Wueest[3], Sonia Tugues[4], Melanie Greter[4], Marianne Böni-Schnetzler[1], Daniel T. Meier[1], Claudia Cavelti-Weder[1,5,6*]

ESM Methods

Hyperinsulinemic-euglycemic clamp studies

Mice were anesthetized with isoflurane and eye ointment was applied to both eyes (Vitamin A, Bausch & Lomb Swiss AG, Switzerland). A catheter (MRE 025, Braintree Scientific, Braintree, MA, USA) was inserted into the right jugular vein and exteriorized at the neck. Five to seven days after the surgery, clamp was performed in freely moving mice after 5 h fasting. To assess basal glucose turnover, mice were infused with 3-[^{3}H] glucose ($3.7*10^4$ bq bolus and 1850 bq/min, Perkin Elmer, Waltham, Massachusetts, USA) for 80 min. After blood collection from the tail vein, hyperinsulinemia was induced by a constant insulin infusion (240 mU/kg bolus and 12 mU/kg*min) paralleled by 3-[^{3}H] glucose infusion ($7.4*10^4$ bq bolus and 3700 bq/min). Steady state glucose infusion rate was calculated once glucose infusion reached a more or less constant rate for 15-20 min with blood glucose levels clamped at ~5-6 mmol/L. Glucose disposal rate was calculated by dividing the rate of 3-[^{3}H] glucose infusion by the plasma 3-[^{3}H] glucose specific activity. Endogenous glucose production during the clamp was calculated by subtracting the glucose infusion rate from the glucose disposal rate. In order to assess tissue specific glucose uptake, a bolus ($3.7*10^5$ bq) of 2-[1-^{14}C] deoxyglucose (Perkin Elmer) was administered via catheter at the end of the steady state period. Blood was sampled 2, 15, 25 and 35 min after bolus delivery. Area under the curve of disappearing plasma 2-[1-^{14}C] deoxyglucose was used together with tissue-concentration of phosphorylated 2-[1-^{14}C] deoxyglucose to calculate glucose uptake. Accumulation of 2-[1-^{14}C] deoxyglucose was determined in an aqueous tissue extract after homogenization. Phosphorylated 2-[1-^{14}C] deoxyglucose was separated by Poly-Prep columns (#731-6212, BioRad, USA).

Isolation and flow cytometry of immune cells

Adipose and liver tissues were minced and digested in a thermomixer (Eppendorf, Germany) using Collagenase IV (Worthington, USA) solution (1HBSS (Gibco, USA), 10mM HEPES (Sigma, USA), 1.5mg/mL Collagenase IV and 8.25µg/mL DNAse I (Roche, Switzerland)) 20-30min, 37 °C with 400rpm. Digestion was stopped by adding FACS buffer (PBS, 0.5% BSA (VWR, USA) and 5mM EDTA (Sigma)), the suspension was filtered (cotton gauze) and washed. Erythrocytes of the adipose tissue were removed by incubation with Red Cell Lysis Buffer (154mM NH4Cl, 10mM KHCO3, 0.1mM EDTA (Gibco, USA), while liver immune cells were enriched by Percoll gradient (GE Healthcare, USA; 70% and 40%, 20min, 600g, minimal brake).

Cells of the peritoneum were isolated by peritoneal lavage with 10mL FACS buffer. Erythrocytes were lysed as described above.

For microglia isolation, whole-brains were mechanically dissociated in FACS buffer using a Dounce-homogenizer (Merck, USA). Cells were passed through a 70µm cell strainer, washed with FACS buffer, and immune cells enriched by Percoll gradient (70% and 37%, 30min, 750g, minimal brake). The microglia-containing interphase was collected, washed and stained.

To isolate colon immune cells, the colon was washed in HBBS, cut into small pieces, washed twice 10 min in HBSS containing 2 mM EDTA (Sigma) shaking at 37 °C, rinsed twice with HBSS and digested with 1mg/mL collagenase VIII (Sigma-Aldrich) and 12.5µg/mL DNase I (Roche), 30min shaking at 37 °C, followed by homogenization (gentleMACS Dissociator: mouse intestine program; Milteny Biotec, Germany). Leukocytes were enriched by Percoll (as described for liver).

Lungs were perfused with PBS, chopped and minced (gentleMACS program m_lung_01-02), digested 30min at 37 °C on shaking with 0.15 WünschU/mg Liberase (Roche)

and 0.1mg/mL DNase I, followed by homogenization using gentleMACS program m_lung_02_01. Leukocytes were enriched by Percoll as described above.

To isolate islets immune cells, pancreas was digested using 1.5mg/mL Collagenase IV (30 min, 37°C) and islets subsequently purified by filtration and hand-picking. Islets were dispersed using 0.01% Trypsin+EDTA (Gibco) (1min 30sec at 37°C).

Spleens were mashed with a syringe plunger on a 70µm filter in red cell lysis buffer, washed and subsequently stained.

Thymus was mashed using a syringe plunger and a 70µm filter in FACS buffer, washed and subsequently stained.

Blood was collected in FACS buffer and stained 30min and lysed using BD FACS lysis solution (BD, USA), according to the manufacturer's protocol.

Cell counting was performed using EVE automated cell counting system (Nano Entek Inc., South Korea), using the following parameters sensitivity 5, size 3-30µm, circularity 50%.

Cell analysis was performed on a FACS LSRII Fortessa (BD). Acquired data were analyzed using FlowJo software (Version 10.6 or higher, TreeStar Inc. Ashland, OR, USA). Gating strategies are summarized in ESM Figures 1 and 2.

Antibodies against CD16/32, CD11b (M1/70), CD11c (N418), MHCII (M5/114.14.2), Ly6C (HK1.4), CD45 (30-F11), F4/80 (BM8), CD103 (2E7), CD24 (M1/69), CD64 (X54-5/7.1), CD3 (145-2C11 and 17A2), CD19 (6D5), NK1.1 (PL136), Ly6G (1A8), CD206 (C068C2), CD25 (PC61), CD4 (GK1.5), CD8 (53-6.7), TCR-b (H57-597), TNF (MP6-XT22), IL10 (JES5-16E3) and IFN (XMG1.2) were purchased from BioLegend (USA). mAb for CCR2 (475301) from R&D (USA), and mAb for Foxp3 (FJK-16s), GATA3 (TWAJ), EOMES (Dan11mag), Il1b (NJTEN3), and IL6 (MP5-20F3) from Thermo Fisher Scientific (USA). mAb for Siglec F (E50-2440) and RORγt (Q31-378) were obtained from BD. Further details ESM Table 1.

For surface staining, single cell suspensions were stained 30min at 4°C. For intracellular staining, the Foxp3 staining kit (Thermo Fischer Scientific) was used according to the manufacturer's protocol. For cytokine staining, cells were cultured 4h in the presence of cellular protein transport inhibitors Brefeldin A (5µg/mL, Sigma) and Monensin (1µg/mL, Sigma) in 2 or 20mM glucose, followed by washing and staining (30 min, 4°C in the dark). Intracellular cytokine staining was performed using IC Fixation Buffer (Thermo Fischer Scientific) according to the manufacturers protocol. Antibody concentrations were verified by titrating experiments.

Beta-cell mass

For heat-induced antigen retrieval, 5µm thick sections were boiled 30min at 93°C in epitope retrieval solution (Biosystems, Switzerland) and stained overnight (4°C) with an insulin antibody (Agilent, USA), washed twice in PBS (5min), stained 2h at room temperature for CD45 (Rat Anti-CD45; 30-F11, BD Bioscience), washed twice with PBS (5min) before applying the secondary antibodies (2h, room temperature; Alexa647 goat anti-guinea pig IgG and Alexa555 goat anti-rat IgG; Thermo Fisher Scientific), then washed twice (PBS), before mounting with a fluorescence mounting media (Dako). Pictures were acquired using a Nikon microscope (4x magnification). For alpha- to beta-cell ratio, slides were stained as described above using primary antibodies against insulin and glucagon (Thermo Fischer Scientitic) and secondary antibodies Alexa488 goat anti-guinea pig IgG and Alexa647 goat anti-rabbit IgG, respectively (Thermo Fischer Scientific). For details on antibodies ESM Table 1.

Images were analyzed using Fiji software. Analysis was performed in a semi-automated way; ilastik software (Version 1.3.2, https://www.ilastik.org/) was trained twice, once to recognize pancreas area excluding background and lymph nodes, and the second time to recognize islets. The masks ilastik generated were used to quantify the areas with Fiji. Beta-

cell mass was defined as the ratio of insulin-positive area to total pancreas area multiplied by the weight of the pancreas. Two sections per animal 100μm apart were quantified.

RNA-Sequencing

A first batch of 9 male mice from different litters of age between 4 and 5 weeks were fed a PLX5622 or a control diet for 5.5. months. A second batch of 4 male mice of age 5 weeks were fed a PLX5622 or control diet for 8.5. months. All mice were euthanized on the same day and RNA isolation was performed on lysed pancreatic islets using a NucleoSpin RNA kit (Macherey Nagel, Germany). RNA samples were quantified and quality-controlled using a High Sensitivity RNA ScreenTape on an Agilent TapeStation instrument. RNA-seq library preparation (Illumina Truseq stranded kit, USA) was performed at the Genomics Facility Basel of the ETH Zurich, Basel, starting from 80ng of total RNA, and controlled on an Agilent Fragment Analyzer instrument. Sequencing was performed on the Illumina NextSeq 500 platform to produce paired-end 38nt reads.

Data analysis was performed by the Bioinformatics Core Facility, Department of Biomedicine, University of Basel. Read quality was assessed with the FastQC tool (version 0.11.5). Reads were mapped to the mouse genome mm10 with STAR (version 2.7.0c)[26] with default parameters, except filtering out multimapping reads with more than 10 alignment locations (outFilterMultimapNmax=10) and filtering reads without evidence in the spliced junction table (outFilterType="BySJout"). All subsequent analyses were performed using the R software (version 4.0.0) and Bioconductor 3.11 packages. The *featureCounts* function from the Bioconductor Rsubread package (version 2.2.3)[27] was used to count the number of reads

(5' ends) overlapping with the exons of each gene (Ensembl release 96) assuming an exon union model[28,29].

The Bioconductor package edgeR (version 3.30.3)[30] was used for differential gene expression analysis. Between samples normalization was performed using the TMM method[31]. Genes with CPM values above 1 in at least 2 samples were retained for the differential expression analysis. To test for gene expression differences between mice fed PLX5622 or a control diet, a model accounting for the treatment and treatment duration effects was fitted to the read counts using a quasi-likelihood testing framework (edgeR functions *glmQLFit* and *glmQLFTest*)[32]. P-values were adjusted by controlling the false discovery rate (FDR; Benjamini-Hochberg method) and genes with a FDR lower than 5% were considered significant. Gene set enrichment analysis was performed with the function camera[33] from the edgeR package (using the default parameter value of 0.01 for the correlations of genes within gene sets) using gene sets from the c5 collection (Gene Ontology categories) and c8 (cell type signatures) of the MSigDB Molecular Signature Database (version 7.2)[34]. We filtered out sets containing less than 10 genes, and gene sets with a FDR lower than 5% were considered significant. A list of mediators of cytokine activity was compiled by downloading the mouse genes annotated to both the GO terms "transcription factor" (GO:0003700) and "regulation of cytokine production" (GO:0001817) using the QuickGO website (download date: May 23rd 2022) and crossed to the differential expression results (ESM Table 4).

ESM Table 1: Reagent and resource table.

REAGENT or RESOURCE	SOURCE	IDENTIFIER
Antibodies		
Anti-mouse CD115 (AFS98)	BioXCell	Cat#BE0213; RRID: AB_2687699
Anti-trinitrophenol rat IgG2a isotype control (2A3)	BioXCell	Cat#BP0089; RRID: AB_1107769
Anti-mouse CD16/32 (93)	Biolegend (USA)	Cat#101321; RRID: AB_2103871
Anti-mouse CD11c (N418) BV650	Biolegend	Cat#117339; RRID: AB_2562414
Anti-mouse CD11c (N418) PE-Cy7	Biolegend	Cat#117318; RRID: AB_493568
Anti-mouse CD11b (M1/70) BV421	Biolegend	Cat#101236; RRID: AB_11203704
Anti-mouse CD45 (30-F11) PerCP-Cy5.5	Biolegend	Cat#103131; RRID: AB_893344
Anti-mouse I-A/I-E (M5/114.15.2) BV785	Biolegend	Cat#107645; RRID: AB_2565977
Anti-mouse Ly6C (HK1.4) FITC	Biolegend	Cat#128005; RRID: AB_1186134
Anti-mouse CCR2 (475301) PE	R&D Systems	Cat#FAB5538P; RRID: AB_10718414
Anti-mouse CD103 (2E7) PE-Dazzle594	Biolegend	Cat#121430; RRID: AB_2566493
Anti-mouse CD24 (M1/69) PE-Cy7	Biolegend	Cat#101821; RRID: AB_756047
Anti-mouse CD64 (X54-5/7.1) APC	Biolegend	Cat#139306; RRID: AB_11219391
Anti-mouse CD3 (145-2C11) APC-Cy7	Biolegend	Cat#100330; RRID: AB_1877170;
Anti-mouse Nk1.1 (PK136) APC-Cy7	Biolegend	Cat#108723; RRID: AB_830870
Anti-mouse CD19 (6D5) APC-Cy7	Biolegend	Cat#115530; RRID: AB_830707
Anti-mouse Siglec F (E50-2440) BV510	BD Biosciences (USA)	Cat#740158; RRID: AB_2739911
Anti-mouse F4/80 (BM8) PE	Biolegend	Cat#123110; RRID: AB_893486
Anti-mouse CD206 (C068C2) A647	Biolegend	Cat#141712; RRID: AB_10900420
Anti-mouse CD3 (17A2) BV711	Biolegend	Cat#100241; RRID:AB_2563945
Anti-mouse TCRβ (H57-597) APC	Biolegend	Cat#109211; RRID:AB_313434
Anti-mouse TCRγδ (GL3) PE	Biolegend	Cat#118107; RRID: AB_313831
Anti-mouse CD8a (53-6.7) A700	Biolegend	Cat#100729; RRID:AB_493702
Anti-mouse CD8b (YTS156.7.7) FITC	Biolegend	Cat#126605; RRID:AB_961293
Anti-mouse CD4 (GK1.5) PE-Dazzle 594	Biolegend	Cat#100456; RRID:AB_2565845
Anti-mouse CD25 (PC61) BV605	Biolegend	Cat#102035; RRID:AB_11126977
Anti-mouse Foxp3 (FJK-16s) APC	Thermo Fisher Scientific	Cat#17-5773-82; RRID:AB_469457
Anti-mouse Rorγ (Q31-378) BV786	BD Biosciences	Cat#564723; RRID:AB_2738916
anti-GATA3 (TWAJ) A488	Thermo Fisher Scientific	Cat#52-9966-43; RRID:AB_2574493
Guinea pig-anti-insulin	Agilent	Cat#A0564; RRID:AB_10013624
Rabbit-anti-glucagon	Thermo Fischer Scientific	Cat#PA-589937; RRID: AB_2805852
Rat Anti-CD45 (30-F11) Monoclonal Antibody, Unconjugated	BD Biosciences	Cat#553076; RRID: AB_394606
Goat anti-Guinea Pig IgG (H+L) Highly Cross-Adsorbed Secondary Antibody, Alexa Fluor 647	Thermo Fisher Scientific	Cat#A-21450; RRID: AB_2735091
Goat anti-Rat IgG (H+L) Cross-Adsorbed Secondary Antibody, Alexa Fluor 555	Thermo Fisher Scientific	Cat#A-21434; RRID:AB_2535855
Goat anti-Guinea Pig IgG (H+L) Highly Cross-Adsorbed Secondary Antibody, Alexa Fluor 488	Thermo Fisher Scientific	Cat#A-11073; RRID:AB_2534117

Goat anti-Rabbit IgG (H+L) Highly Cross-Adsorbed Secondary Antibody, Alexa Fluor 647	Thrmo Fischer Scientific	Cat#A-21244; AB_2535812
**Chemicals, Peptides, and Recombinant Proteins**		
Recombinant Il-1β	R&D Systems	Cat#4001-ML
Collagenase IV	Worthington	Cat# LS004189
Collagenase VIII	Sigma-Aldrich	Cat# C2139
Liberase	Roche	Cat# 5401020001
DNase 1	Roche	Cat# 11284932001
Percoll	GE Healthcare (USA)	Cat# GE17-0891-01
PLX5622 (CFS1R inhibitor)	Plexxikon	MTA
**Critical Commercial Assays**		
Mouse/rat insulin kit	MesoScale Diagnostics	Cat#K152BZC
V-Plex custom mouse cytokine proinflammatory panel 1 mouse TNF-α and mouse IL-6	MesoScale Diagnostics	Cat#K15048
Foxp3 staining Kit	Thermo Fisher Scientific	Cat# 00-5523-00
NucleoSpin RNA kit	Macherey Nagel	Cat# 740955
RNeasy Plus Universal Mini kit	Qiagen	Cat# 73404
GoScript™	Promega	Cat# A5003
GoTaq qPCR Master Mix	Promega	Cat# A4472919
**Deposited Data**		
scRNA-seq Data	This paper	GSE189434
**Experimental Models: Organisms/Strains**		
Mouse, C57BL/6NCrl	Charles River laboratories (Germany)	RRID:IMSR_CRL:027
**Oligonucleotides**		
Primers for qPCR -> ESM Table 2	Microsynth	N/A
**Software and Algorithms**		
Fiji software ImageJ 1.52n with Java 1.8.0_172		https://imagej.net/Fiji
Ilastik (version 1.3.2)		www.ilastik.org
Flow jo (version 9.9 or higher)	Becton Dickinson & Company (BD)	https://flowjo.com
BD FACS Diva (version 8.0.1)	Becton Dickinson & Company (BD)	https://www.bdbiosciences.com/en-us/instruments/research-instruments/research-software/flow-cytometry-acquisition/facsdiva-software
Prism 8	GraphPad Software, LLc.	https://www.graphpad.com
R version 3.6	The R Foundation	https://www.r-project.org

ESM Table 2: Primers sequences used for quantitative real time-PCR.

Gene	Forward Primer	Reverse Primer
**Housekeeping genes**		
B2m	5′ TTCTGGTGCTTGTCTCACTGA	5′ CAGTATGTTCGGCTTCCCATTC
Ppia	5′ GAGCTGTTTGCAGACAAAGTTC	5′ CCCTGGCACATGAATCCTGG
**Inflammation markers**		
Tnf	5′ ACTGAACTTCGGGGTGATCG	5′ TGAGGGTCTGGGCCATAGAA
Il1b	5′ GCAACTGTTCCTGAACTCAACT	5′ ATCTTTTGGGGTCCGTCAACT
Il6	5′ GGATACCACTCCCAACAGACCT	5′ GCCATTGCACAACTCTTTTCTC
Cxcl1	5′ CTGGGATTCACCTCAAGAACATC	5′ CAGGGTCAAGGCAAGCCTC
**Immune cells**		
Cd68	5′ GCAGCACAGTGGACATTCAT	5′ AGAGAAACATGGCCC GAAGT
Adgre1 (Emr1)	5′ GCC CAG GAGTGGAATGTCAA	5′ CAGACACTCATCAACATCTGCG
CD45	5’ATGGTCCTCTGAATAAAGCCCA	5’TCAGCACTATTGGTAGGCTCC
**Beta-cell identity**		
Pdx1	5′CCC CAG TTT ACA AGC TCG CT	5′CTC GGT TCC ATT CGG GAA AGG
Foxo1	5′GTA CGC CGA CCT CAT CAC CA	5′TGC TGT CGC CCT TAT CCT TG
Ins2	5′CCC TGC TGG CCC TGC TCT T	5′AGG TCT GAA GGT CAC CTG CT

ESM Table 3: Differential gene expression of islets upon CSF1R-inhibition by PLX5622 (separate excel file). Full results table for the differential expression analysis comparing islets isolated from mice on PLX5622 or control diet (first tab).

ESM Table 4: Gene set enrichment analysis based on the differential expression analysis, performed on the MSigDB collection c5 (separate excel file). Full results from the gene set enrichment analysis based on the differential expression analysis, performed on the MSigDB collection c5 (second tab).

ESM Table 5: Gene set enrichment analysis based on the differential expression analysis, performed on the MSigDB collection c8 (separate excel file).

Full results from the gene set enrichment analysis based on the differential expression analysis, performed on the MSigDB collection c8 (third tab).

ESM Table 6: Gene set enrichment analysis based on the differential expression analysis, performed on the hallmark pathways (separate excel file). Full results from the gene set enrichment analysis based on the differential expression analysis, performed on the hallmark pathways (fourth tab).

ESM Table 7: Down-regulated mediators of cytokine activity upon CSF1R-inhibition.

Symbol	Description	GeneID	logFC	logCPM	FDR
Lilrb4a	**Leukocyte immunoglobulin-like receptor, subfamily B, member 4A**	ENSMUSG00000112148	-4.411	1.761	0.00000005
Cd74	**CD74 antigen (invariant polypeptide of major histocompatibility complex, class II antigen-associated)**	ENSMUSG00000024610	-4.910	5.579	0.00000005
Mmp12	**Matrix metallopeptidase 12**	ENSMUSG00000049723	-4.854	2.173	0.0000001
Nckap1l	**NCK associated protein 1 like**	ENSMUSG00000022488	-3.412	0.753	0.0000001
Cybb	**Cytochrome b-245, beta polypeptide**	ENSMUSG00000015340	-3.673	0.725	0.0000002
Laptm5	**Lysosomal-associated protein transmembrane 5**	ENSMUSG00000028581	-3.198	1.243	0.0000002
Tyrobp	**TYRO protein tyrosine kinase binding protein**	ENSMUSG00000030579	-3.143	0.072	0.0000004
Ptafr	**Platelet-activating factor receptor**	ENSMUSG00000056529	-2.271	-0.670	0.000002
C3ar1	**Complement component 3a receptor 1**	ENSMUSG00000040552	-2.832	-0.292	0.000003
Tnf	**Tumor necrosis factor**	ENSMUSG00000024401	-2.095	-1.191	0.000004
Cd36	**CD36 molecule**	ENSMUSG00000002944	-1.652	2.075	0.000004
Slc11a1	**Solute carrier family 11 (proton-coupled divalent metal ion transporters), member 1**	ENSMUSG00000026177	-2.315	0.219	0.000005
Nlrp3	**NLR family, pyrin domain containing 3**	ENSMUSG00000032691	-2.305	-0.653	0.00001
Spi1	**Spleen focus forming virus (SFFV) proviral integration oncogene**	ENSMUSG00000002111	-2.221	-0.889	0.00001
Ifi204	**Interferon activated gene 204**	ENSMUSG00000073489	-1.971	-0.138	0.00002
Ptprc	**Protein tyrosine phosphatase, receptor type, C**	ENSMUSG00000026395	-2.541	-0.168	0.00003
Cd83	**CD83 antigen**	ENSMUSG00000015396	-1.325	1.355	0.00004
Ifi207	**Interferon activated gene 207**	ENSMUSG00000073490	-1.904	0.440	0.00004
Ccr5	**Chemokine (C-C motif) receptor 5**	ENSMUSG00000079227	-2.322	-0.932	0.0001
Ccl3	**Chemokine (C-C motif) ligand 3**	ENSMUSG00000000982	-2.003	-1.437	0.0001
Acp5	**Acid phosphatase 5, tartrate resistant**	ENSMUSG00000001348	-2.603	-0.524	0.0001
Clec7a	**C-type lectin domain family 7, member a**	ENSMUSG00000079293	-2.368	-0.785	0.0001
Rab7b	**RAB7B, member RAS oncogene family**	ENSMUSG00000052688	-1.335	0.041	0.0001
Tnfrsf1b	**Tumor necrosis factor receptor superfamily, member 1b**	ENSMUSG00000028599	-1.192	1.974	0.0001
Havcr2	**Hepatitis A virus cellular receptor 2**	ENSMUSG00000020399	-2.028	-0.137	0.0001
Axl	**AXL receptor tyrosine kinase**	ENSMUSG00000002602	-0.919	2.929	0.0001
Vsir	**V-set immunoregulatory receptor**	ENSMUSG00000020101	-1.032	0.310	0.0002
Hmox1	**Heme oxygenase 1**	ENSMUSG00000005413	-0.868	5.188	0.0003
Fcgr3	**Fc receptor, igg, low affinity III**	ENSMUSG00000059498	-1.965	-1.002	0.0004
Cebpa	**CCAAT/enhancer binding protein (C/EBP), alpha**	ENSMUSG00000034957	-0.988	0.635	0.0005
Irf5	**Interferon regulatory factor 5**	ENSMUSG00000029771	-1.474	-0.277	0.001

Syk	**Spleen tyrosine kinase**	ENSMUSG00000021457	-1.661	0.541	0.001
Bst2	**Bone marrow stromal cell antigen 2**	ENSMUSG00000046718	-0.876	2.108	0.002
Stat6	**Signal transducer and activator of transcription 6**	ENSMUSG00000002147	-0.337	5.483	0.003
Pld4	**Phospholipase D family, member 4**	ENSMUSG00000052160	-1.210	-0.095	0.003
Cd14	**CD14 antigen**	ENSMUSG00000051439	-1.234	2.233	0.003
Sp100	**Nuclear antigen Sp100**	ENSMUSG00000026222	-1.123	0.562	0.004
Tlr2	**Toll-like receptor 2**	ENSMUSG00000027995	-0.387	5.503	0.005
Cyba	**Cytochrome b-245, alpha polypeptide**	ENSMUSG00000006519	-0.958	0.640	0.007
Ccl5	**Chemokine (C-C motif) ligand 5**	ENSMUSG00000035042	-1.891	-0.134	0.007
P2rx7	**Purinergic receptor P2X, ligand-gated ion channel, 7**	ENSMUSG00000029468	-1.002	0.010	0.009
Fcer1g	**Fc receptor, ige, high affinity I, gamma polypeptide**	ENSMUSG00000058715	-0.667	1.924	0.011
Npas4	**Neuronal PAS domain protein 4**	ENSMUSG00000045903	0.722	7.356	0.012
Zeb2	**Zinc finger E-box binding homeobox 2**	ENSMUSG00000026872	-0.600	1.836	0.012
Il1r2	**Interleukin 1 receptor, type II**	ENSMUSG00000026073	-0.727	1.623	0.017
Oas1a	**2'-5' oligoadenylate synthetase 1A**	ENSMUSG00000052776	-0.821	1.078	0.020
Zbtb48	**Zinc finger and BTB domain containing 48**	ENSMUSG00000028952	0.313	3.950	0.021
Irf7	**Interferon regulatory factor 7**	ENSMUSG00000025498	-0.985	2.854	0.021
Gbp5	**Guanylate binding protein 5**	ENSMUSG00000105504	-1.286	0.583	0.023
Hsf2	**Heat shock factor 2**	ENSMUSG00000019878	0.251	4.850	0.023
Cd40	**CD40 antigen**	ENSMUSG00000017652	-0.921	0.781	0.027
Kit	**KIT proto-oncogene receptor tyrosine kinase**	ENSMUSG00000005672	-0.463	2.253	0.029
Trim30a	**Tripartite motif-containing 30A**	ENSMUSG00000030921	-1.067	-0.048	0.029
Zfp9	**Zinc finger protein 9**	ENSMUSG00000072623	0.300	6.222	0.031
Akap8	**A kinase (PRKA) anchor protein 8**	ENSMUSG00000024045	0.279	6.246	0.041
Frmd8	**FERM domain containing 8**	ENSMUSG00000024816	-0.354	3.274	0.048
Gprc5b	**G protein-coupled receptor, family C, group 5, member B**	ENSMUSG00000008734	-0.336	3.524	0.048

ESM Figures

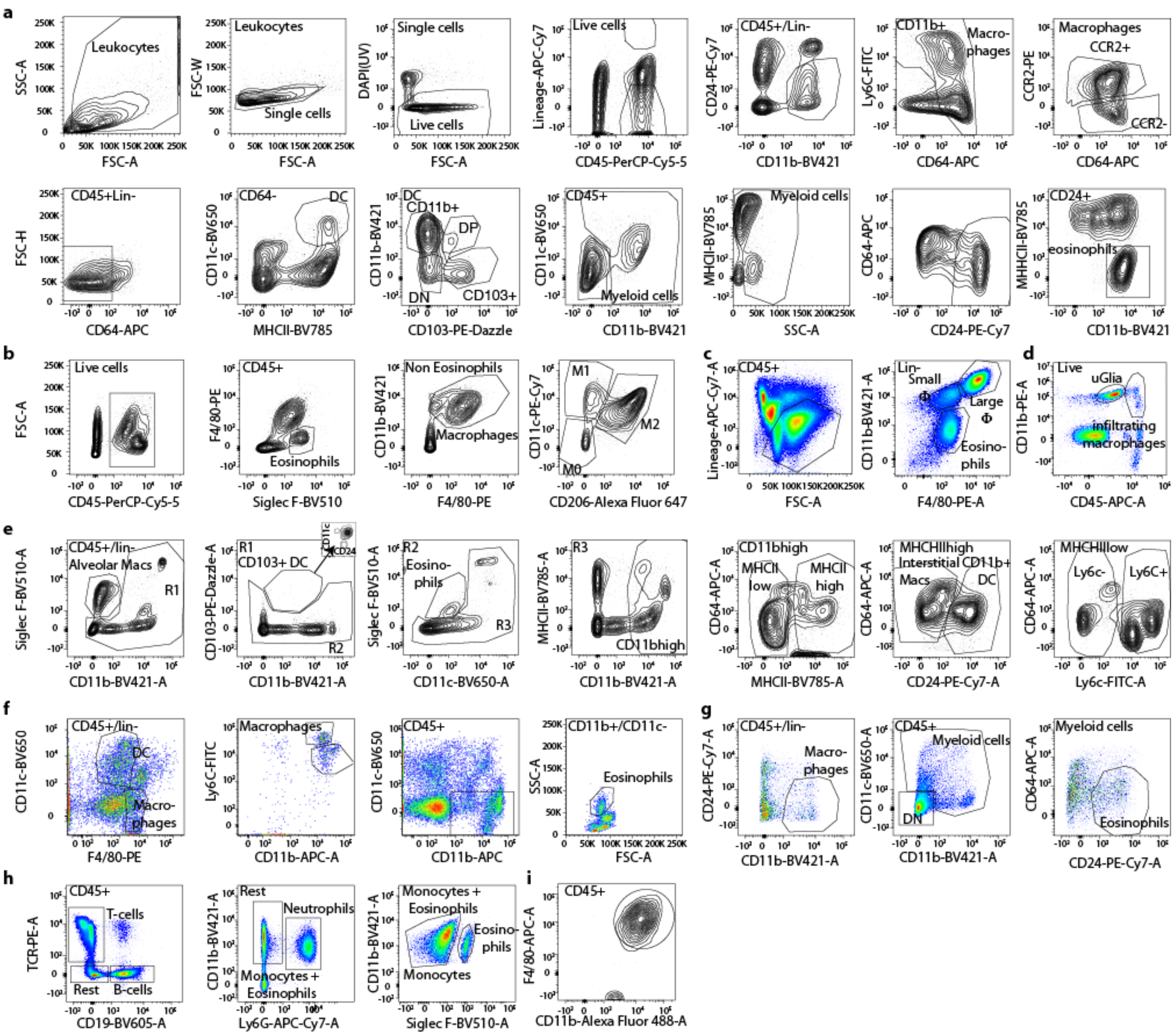

ESM Fig. 1: Gating strategies of macrophages, dendritic cells and eosinophils in different organs. Gating strategy in (**a**) colon (**b**) adipose tissue, (**c**) peritoneum, (**d**) brain (**e**) lung, (**f**) liver, (**g**) spleen, (**h**) blood and (**i**) islets. All gating strategies included a leukocyte gate, followed by doublet exclusion and exclusion of dead cells (only shown in panel a). Further gating included CD45 and lineage (CD19, Nk1.1, CD3 and Ly6G).

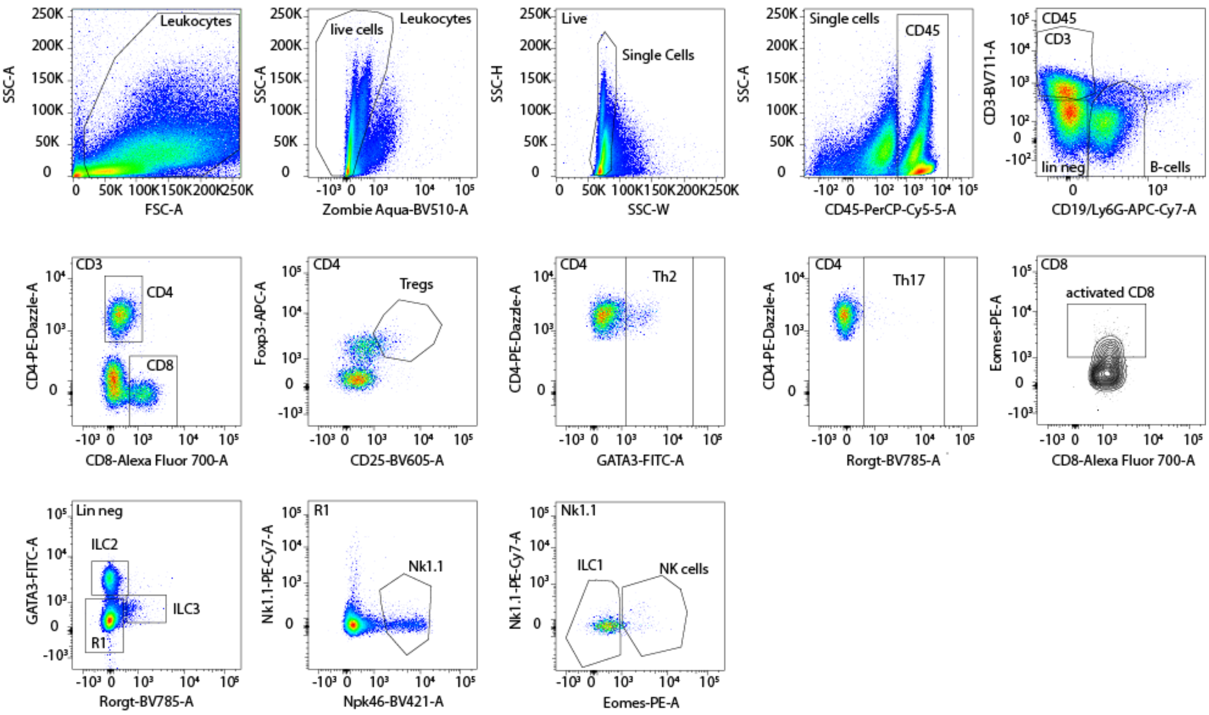

ESM Fig. 2: Gating strategy for Innate lymphoid cells and adaptive immunity. Leukocytes were gated for live cells, and doublets excluded by FSC W and H. CD45^{+} cells were analyzed for T and B-cells and ILCs. T-cells were defined as CD3^{+} and either CD8^{+} and CD4^{+}, the latter ones were further analyzed for Tregs defined as Foxp3^{+} and CD25^{+}, Th2 identified by the expression of GATA3 and TH17 identified by RORγt. ILCs were defied as CD3^{-}/lineage^{-} cells, different subpopulations were further defined as RORγt^{+} (ILC3), and GATA3^{+} (ILC2).

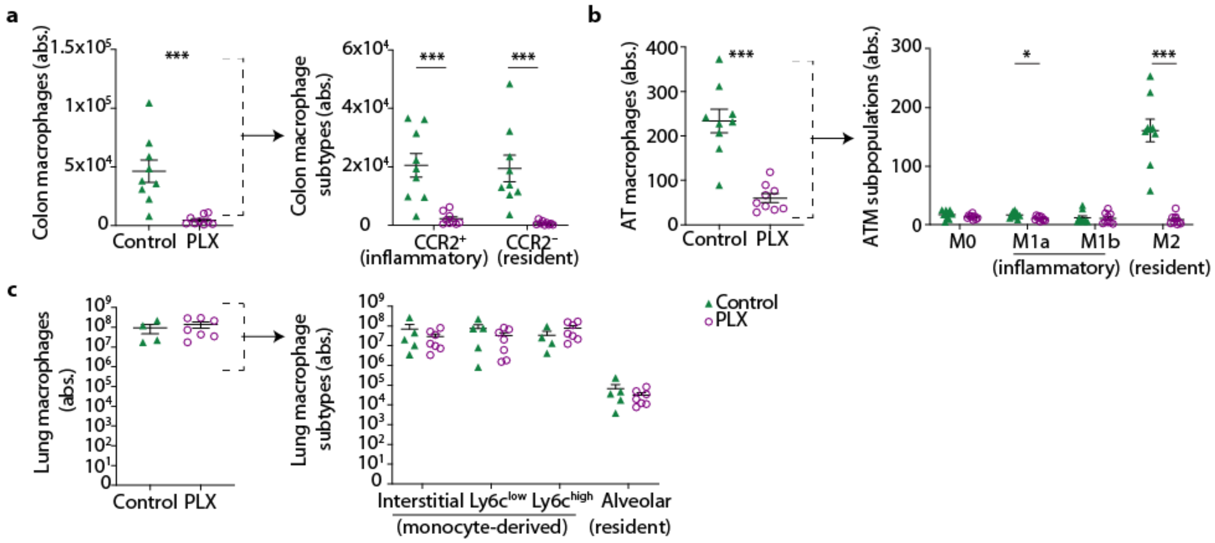

ESM Fig. 3: Absolute numbers of tissue resident macrophages upon PLX5622 treatment. Set-up see Fig. 1. **a**-**c**, Total macrophages (left panel) and subpopulations (right panel) of (**a**) colon, (**b**) adipose tissue and (**c**) lung. Data are shown as mean±SEM. Data are representative of 3 (**a, b**) independent experiments or one experiment (**c**) with each data point representing an individual mouse. *p<0.05, **p<0.01, ***p<0.001, unpaired Mann-Whitney U test with two-tailed distribution.

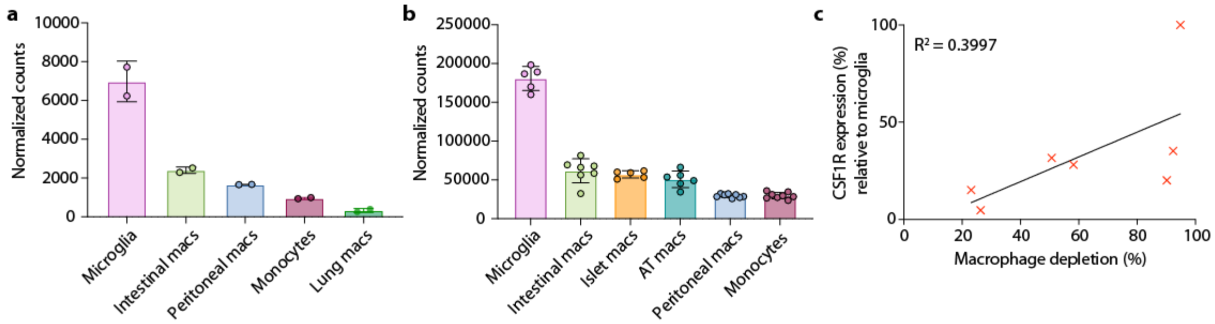

ESM Fig. 4: CSF1R expression in macrophages across different organs. (**a**) Dataset obtained from Lavin, 2014. (**b**) Data obtained from Brykcznska, 2020. (**c**) Correlation between macrophage depletion (%) and CSF1R expression in relation to CSF1R expression in microglia.

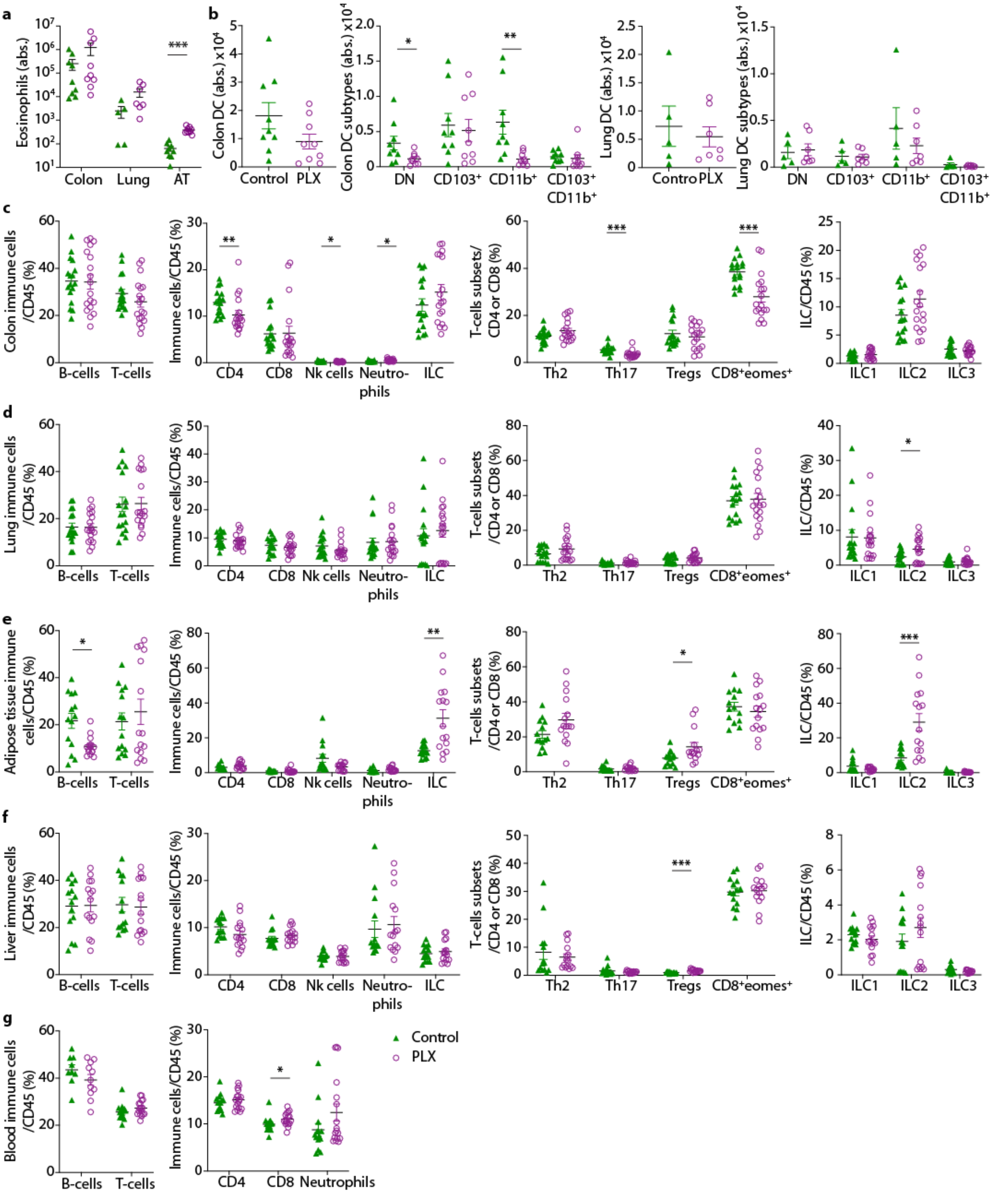

ESM Fig. 5: Changes in immune cells upon CSF1R-inhibition by PLX5622. Mice were fed a PLX5622-containing (purple circles) or control diet (green triangles) starting from 4-5 weeks of age for up to 5.5 months (**a, b**) or starting from 8-9 weeks of age for 3 weeks (**c-g**). **a**, Absolute numbers of eosinophils. **b**, Absolute numbers of dendritic cells (DCs) und their subpopulations in the colon and lung. **c-f**, Frequencies of B and T-cells, CD4, CD8 T-cells and

their subsets, NK cells, neutrophils and ILC in colon (**c**), lung (**d**), adipose tissue (**e**) and liver (**f**). **g**, Frequencies of B and T cells and neutrophils in blood. Data are shown as mean±SEM. Pooled data of three independent experiments (**c**-**g**) or representative of three or one experiment (**a, b**) with each data point representing an individual mouse. *$p<0.05$, **$p<0.01$, ***$p<0.001$, unpaired Mann-Whitney U test with two-tailed distribution.

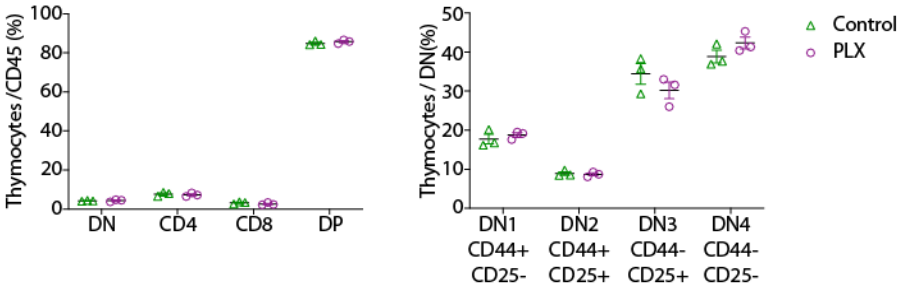

ESM Fig. 6: Depletion of macrophages does not affect T cell development. Mice were fed a PLX5622-containing (purple circles) or control diet (green triangles) for 3 weeks. Total thymocytes (left panel) and double negative thymocytes (right panel). Data are shown as mean±SEM. One experiment with 3 mice each, each data point representing an individual mouse.

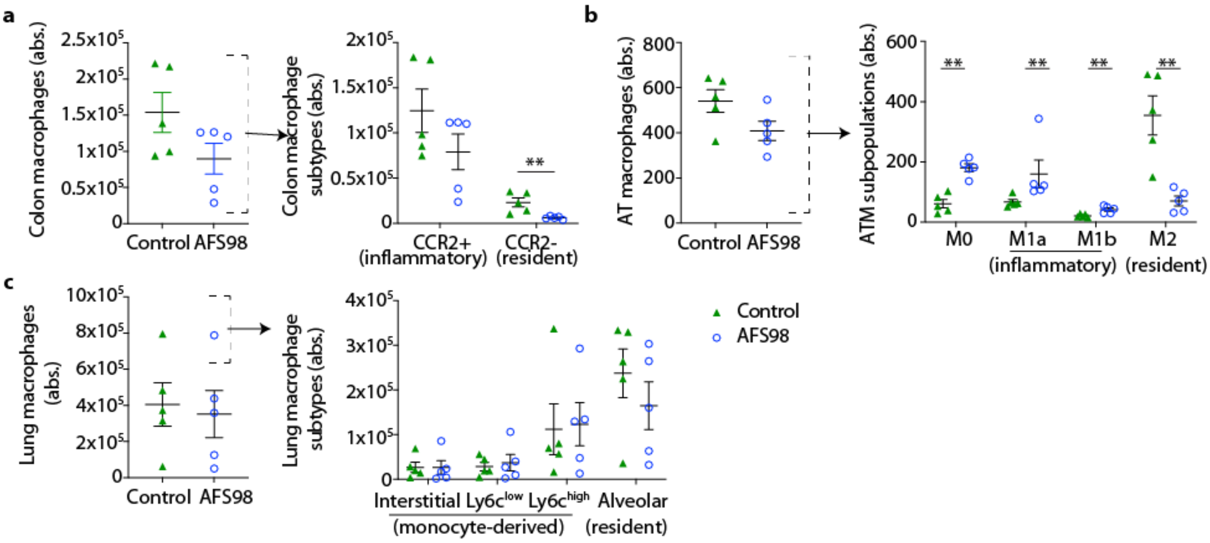

ESM Fig. 7: Absolute numbers of tissue resident macrophages upon CSF1R antibody treatment. Mice were treated for three weeks with the CSF1R antibody AFS98 (blue circles) or a rat IgG2a isotype control (green triangles) and immune cells of different organs were assessed by flow **a-c**, Total macrophages (left panel) and subpopulations (right panel) of (**a**) colon, (**b**) adipose tissue and (**c**) lung. Data are shown as mean±SEM. One experiments, with 5 mice each, each data point representing an individual mouse. **p<0.01, unpaired Mann-Whitney U test with two-tailed distribution.

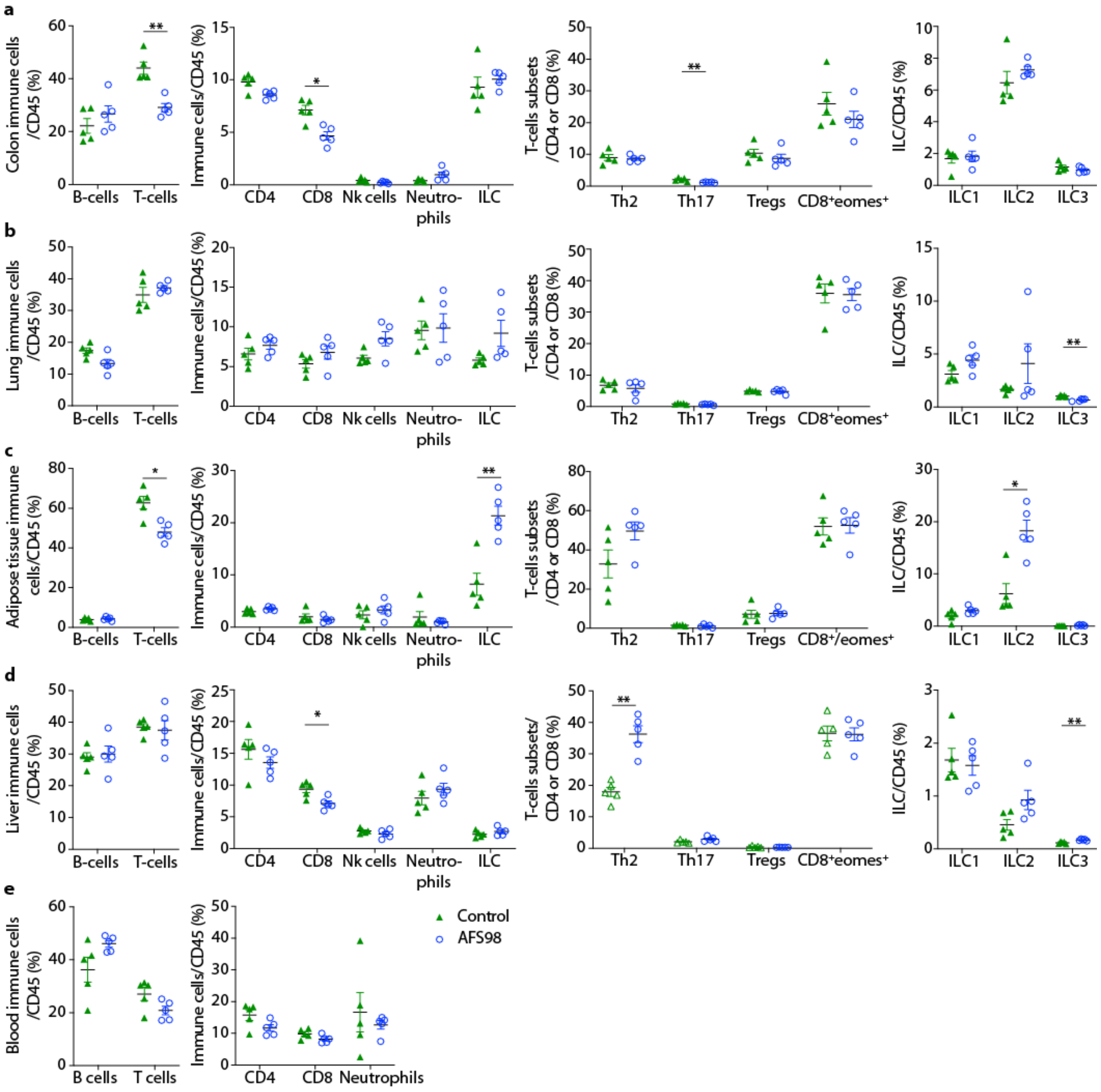

ESM Fig. 8: Changes in immune cells upon CSF1R antibody treatment. Mice were treated for three weeks with the CSF1R antibody AFS98 (blue circles) or a rat IgG2a isotype control (green triangles). **a-d**, Frequencies of B and T-cells, CD4, CD8 T-cells and their subsets, NK cells, neutrophils and ILCs in colon (**a**), lung (**b**), adipose tissue (**c**) and liver (**d**). **e**, Frequencies of B and T cells and neutrophils in blood. Data are shown as mean±SEM. One experiments, with 5 mice each, each data point representing an individual mouse. *p<0.05, **p<0.01, unpaired Mann-Whitney U test with two-tailed distribution.

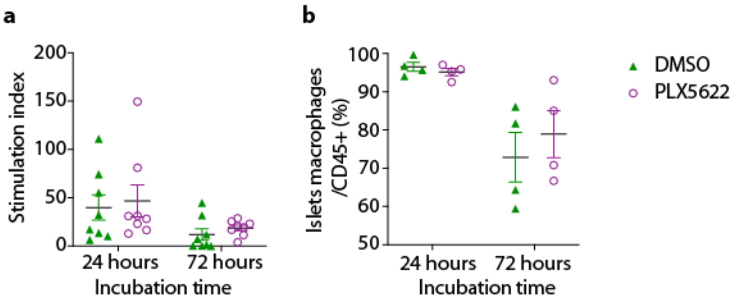

ESM Fig. 9: PLX5622 is not toxic to pancreatic islets isolated from wild-type mice. *Ex-vivo* treatment of islets with PLX5622 (purple circles) or DMSO (green triangles) for 24 and 72 hours, respectively. **a,b,** Effect of 24 or 72 hours of PLX5622 treatment on (**a**) stimulation index during *ex vivo* glucose stimulated insulin secretion (GSIS) and (**b**) islet macrophages. Data are shown as mean±SEM. Pooled data from two independent experiments, unpaired Mann-Whitney U test with two-tailed distribution.

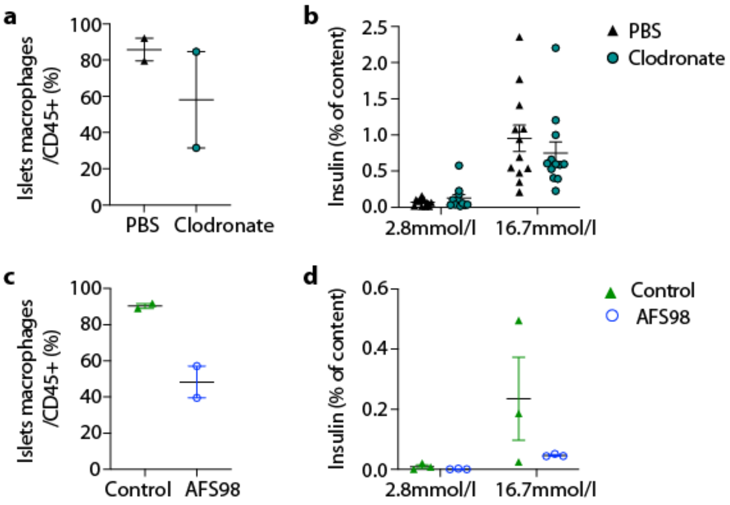

ESM Fig. 10: Macrophage depletion reduces insulin secretion in wild type islets. Islet macrophages were depleted either *ex-vivo* by clodronate treatment for 24 hours or by AFS98 antibody (i.p. 1mg/mouse) once weekly over 3 weeks. **a,b** Macrophages were depleted by clodronate (turquoise circles) or PBS liposomes (control; black triangles) treatment over 24 hours. (**a**) Frequency of islet macrophages after 24 hours of culture with clodronate or PBS liposomes. (**b**) *Ex-vivo* insulin release in a glucose stimulated insulin secretion assay (GSIS) after *ex-vivo* treatment of islets with clodronate or PBS liposome. **c, d** Mice were treated over 3 weeks once weekly with the CSF1R anti-body AFS98 (blue circles) or its corresponding control anti-body (green triangles) at a dose of 1mg/mouse (i.p.). (**c**) Frequency of islet macrophages after treatment. (**d**) Insulin release *ex-vivo* in a glucose stimulated insulin secretion assay (GSIS) over one hour of islets from treated mice. Data are shown as mean±SEM, Pooled data from two independent experiments (b), three independent experiment (c) or one experiment (c, d), with each point representing one mouse (d) or technical replicates (b) unpaired Mann-Whitney U test with two-tailed distribution.

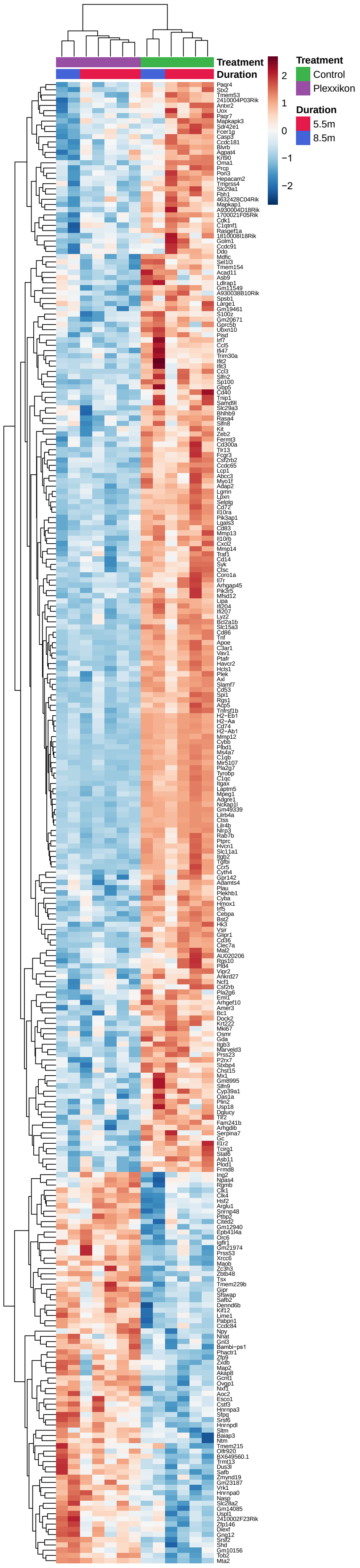
Treatment
Duration
2
1
0
−1
−2
Treatment
Control
Plexxikon
Duration
5.5m
8.5m

ESM Fig. 11: Heatmap showing the centered and scaled normalized expression levels of genes differentially expressed in islets of Langerhans upon CSF1R-inhibition. FDR<5%

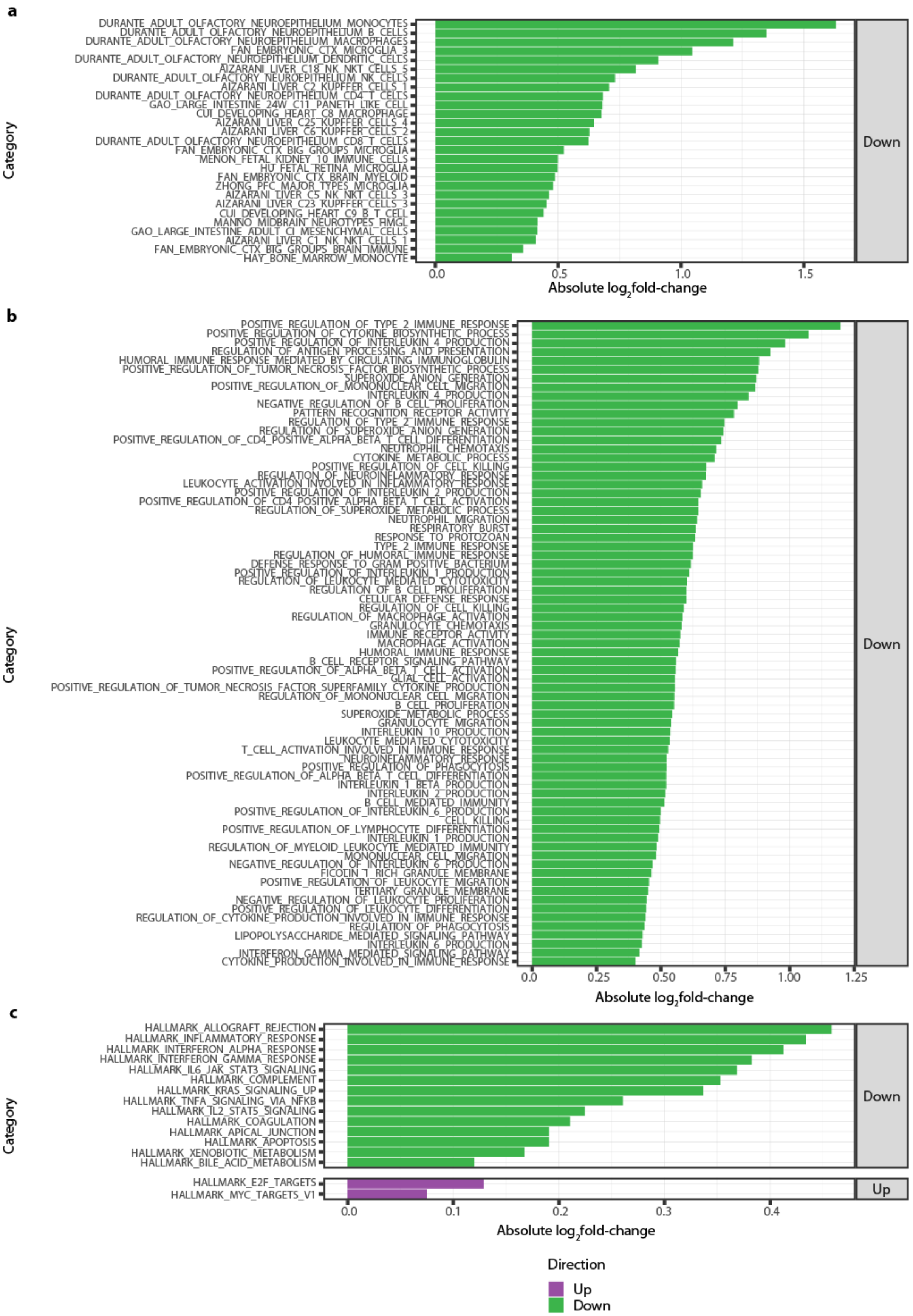

a
Category
DURANTE_ADULT_OLFACTORY_NEUROEPITHELIUM_MONOCYTES
DURANTE_ADULT_OLFACTORY_NEUROEPITHELIUM_B_CELLS
DURANTE_ADULT_OLFACTORY_NEUROEPITHELIUM_MACROPHAGES
FAN_EMBRYONIC_CTX_MICROGLIA_3
DURANTE_ADULT_OLFACTORY_NEUROEPITHELIUM_DENDRITIC_CELLS
AIZARANI_LIVER_C18_NK_NKT_CELLS_5
DURANTE_ADULT_OLFACTORY_NEUROEPITHELIUM_NK_CELLS
AIZARANI_LIVER_C2_KUPFFER_CELLS_1
DURANTE_ADULT_OLFACTORY_NEUROEPITHELIUM_CD4_T_CELLS
GAO_LARGE_INTESTINE_24W_C11_PANETH_LIKE_CELL
CUI_DEVELOPING_HEART_C8_MACROPHAGE
AIZARANI_LIVER_C25_KUPFFER_CELLS_4
AIZARANI_LIVER_C6_KUPFFER_CELLS_2
DURANTE_ADULT_OLFACTORY_NEUROEPITHELIUM_CD8_T_CELLS
FAN_EMBRYONIC_CTX_BIG_GROUPS_MICROGLIA
MENON_FETAL_KIDNEY_10_IMMUNE_CELLS
HU_FETAL_RETINA_MICROGLIA
FAN_EMBRYONIC_CTX_BRAIN_MYELOID
ZHONG_PFC_MAJOR_TYPES_MICROGLIA
AIZARANI_LIVER_C5_NK_NKT_CELLS_3
AIZARANI_LIVER_C23_KUPFFER_CELLS_3
CUI_DEVELOPING_HEART_C9_B_T_CELL
MANNO_MIDBRAIN_NEUROTYPES_HMGL
GAO_LARGE_INTESTINE_ADULT_CI_MESENCHYMAL_CELLS
AIZARANI_LIVER_C1_NK_NKT_CELLS_1
FAN_EMBRYONIC_CTX_BIG_GROUPS_BRAIN_IMMUNE
HAY_BONE_MARROW_MONOCYTE
Down
0.0
0.5
1.0
1.5
Absolute log2fold-change
b
Category
POSITIVE_REGULATION_OF_TYPE_2_IMMUNE_RESPONSE
POSITIVE_REGULATION_OF_CYTOKINE_BIOSYNTHETIC_PROCESS
POSITIVE_REGULATION_OF_INTERLEUKIN_4_PRODUCTION
REGULATION_OF_ANTIGEN_PROCESSING_AND_PRESENTATION
HUMORAL_IMMUNE_RESPONSE_MEDIATED_BY_CIRCULATING_IMMUNOGLOBULIN
POSITIVE_REGULATION_OF_TUMOR_NECROSIS_FACTOR_BIOSYNTHETIC_PROCESS
SUPEROXIDE_ANION_GENERATION
POSITIVE_REGULATION_OF_MONONUCLEAR_CELL_MIGRATION
INTERLEUKIN_4_PRODUCTION
NEGATIVE_REGULATION_OF_B_CELL_PROLIFERATION
PATTERN_RECOGNITION_RECEPTOR_ACTIVITY
REGULATION_OF_TYPE_2_IMMUNE_RESPONSE
REGULATION_OF_SUPEROXIDE_ANION_GENERATION
POSITIVE_REGULATION_OF_CD4_POSITIVE_ALPHA_BETA_T_CELL_DIFFERENTIATION
NEUTROPHIL_CHEMOTAXIS
CYTOKINE_METABOLIC_PROCESS
POSITIVE_REGULATION_OF_CELL_KILLING
REGULATION_OF_NEUROINFLAMMATORY_RESPONSE
LEUKOCYTE_ACTIVATION_INVOLVED_IN_INFLAMMATORY_RESPONSE
POSITIVE_REGULATION_OF_INTERLEUKIN_2_PRODUCTION
POSITIVE_REGULATION_OF_CD4_POSITIVE_ALPHA_BETA_T_CELL_ACTIVATION
REGULATION_OF_SUPEROXIDE_METABOLIC_PROCESS
NEUTROPHIL_MIGRATION
RESPIRATORY_BURST
RESPONSE_TO_PROTOZOAN
TYPE_2_IMMUNE_RESPONSE
REGULATION_OF_HUMORAL_IMMUNE_RESPONSE
DEFENSE_RESPONSE_TO_GRAM_POSITIVE_BACTERIUM
POSITIVE_REGULATION_OF_INTERLEUKIN_1_PRODUCTION
REGULATION_OF_LEUKOCYTE_MEDIATED_CYTOTOXICITY
REGULATION_OF_B_CELL_PROLIFERATION
CELLULAR_DEFENSE_RESPONSE
REGULATION_OF_CELL_KILLING
REGULATION_OF_MACROPHAGE_ACTIVATION
GRANULOCYTE_CHEMOTAXIS
IMMUNE_RECEPTOR_ACTIVITY
MACROPHAGE_ACTIVATION
HUMORAL_IMMUNE_RESPONSE
B_CELL_RECEPTOR_SIGNALING_PATHWAY
POSITIVE_REGULATION_OF_ALPHA_BETA_T_CELL_ACTIVATION
GLIAL_CELL_ACTIVATION
POSITIVE_REGULATION_OF_TUMOR_NECROSIS_FACTOR_SUPERFAMILY_CYTOKINE_PRODUCTION
REGULATION_OF_MONONUCLEAR_CELL_MIGRATION
B_CELL_PROLIFERATION
SUPEROXIDE_METABOLIC_PROCESS
GRANULOCYTE_MIGRATION
INTERLEUKIN_10_PRODUCTION
LEUKOCYTE_MEDIATED_CYTOTOXICITY
T_CELL_ACTIVATION_INVOLVED_IN_IMMUNE_RESPONSE
NEUROINFLAMMATORY_RESPONSE
POSITIVE_REGULATION_OF_PHAGOCYTOSIS
POSITIVE_REGULATION_OF_ALPHA_BETA_T_CELL_DIFFERENTIATION
INTERLEUKIN_1_BETA_PRODUCTION
INTERLEUKIN_2_PRODUCTION
B_CELL_MEDIATED_IMMUNITY
POSITIVE_REGULATION_OF_INTERLEUKIN_6_PRODUCTION
CELL_KILLING
POSITIVE_REGULATION_OF_LYMPHOCYTE_DIFFERENTIATION
INTERLEUKIN_1_PRODUCTION
REGULATION_OF_MYELOID_LEUKOCYTE_MEDIATED_IMMUNITY
MONONUCLEAR_CELL_MIGRATION
NEGATIVE_REGULATION_OF_INTERLEUKIN_6_PRODUCTION
FICOLIN_1_RICH_GRANULE_MEMBRANE
POSITIVE_REGULATION_OF_LEUKOCYTE_MIGRATION
TERTIARY_GRANULE_MEMBRANE
NEGATIVE_REGULATION_OF_LEUKOCYTE_PROLIFERATION
POSITIVE_REGULATION_OF_LEUKOCYTE_DIFFERENTIATION
REGULATION_OF_CYTOKINE_PRODUCTION_INVOLVED_IN_IMMUNE_RESPONSE
REGULATION_OF_PHAGOCYTOSIS
LIPOPOLYSACCHARIDE_MEDIATED_SIGNALING_PATHWAY
INTERLEUKIN_6_PRODUCTION
INTERFERON_GAMMA_MEDIATED_SIGNALING_PATHWAY
CYTOKINE_PRODUCTION_INVOLVED_IN_IMMUNE_RESPONSE
Down
0.0
0.25
0.50
0.75
1.00
1.25
Absolute log2fold-change
c
Category
HALLMARK_ALLOGRAFT_REJECTION
HALLMARK_INFLAMMATORY_RESPONSE
HALLMARK_INTERFERON_ALPHA_RESPONSE
HALLMARK_INTERFERON_GAMMA_RESPONSE
HALLMARK_IL6_JAK_STAT3_SIGNALING
HALLMARK_COMPLEMENT
HALLMARK_KRAS_SIGNALING_UP
HALLMARK_TNFA_SIGNALING_VIA_NFKB
HALLMARK_IL2_STAT5_SIGNALING
HALLMARK_COAGULATION
HALLMARK_APICAL_JUNCTION
HALLMARK_APOPTOSIS
HALLMARK_XENOBIOTIC_METABOLISM
HALLMARK_BILE_ACID_METABOLISM
Down
HALLMARK_E2F_TARGETS
HALLMARK_MYC_TARGETS_V1
Up
0.0
0.1
0.2
0.3
0.4
Absolute log2fold-change
Direction
Up
Down

ESM Fig 12: Top differentially regulated MSigDB signatures upon CSF1R-inhhibition. **(a)** Top differentially regulated MSigDB C8 cell type signatures upon CSF1R-inhibition**.** p-value < 10^{-10}. **(b)** Top differentially regulated MSigDB C5 gene ontology terms upon CSF1R-inhibition. p-value < 10^{-10} and gene set size < 100 genes. **(c)** Top differentially regulated MSigDB hallmark gene sets upon CSF1R-inhibition. FDR<5%. Full analysis results in ESM Table 3.
